# Biochemical Reduction of Metal Salts as a Prominent Approach for Biohybrid Nanomaterials Production: A Review

**DOI:** 10.3390/nano15241899

**Published:** 2025-12-17

**Authors:** Daniil A. Bogachikhin, Marina A. Abramkina, Anastasia K. Dzuba, Bogdan Ya. Karlinskii, Vyacheslav A. Arlyapov

**Affiliations:** 1 BioChemTech Research Center, Tula State University, Lenin pr. 92, 300012 Tula, Russia; d.a.bogachihin@tsu.tula.ru (D.A.B.); abramkinamarina2@gmail.com (M.A.A.); polunina99@list.ru (A.K.D.); 2 Zelinsky Institute of Organic Chemistry, Russian Academy of Sciences, Leninsky pr. 47, 119991 Moscow, Russia

**Keywords:** biohybrid materials, nanobiotechnology, metal nanoparticles, sustainable chemistry

## Abstract

Metal nanoparticles are unique materials with diverse properties and a wide range of paramount applications in various scientific fields, from catalysis and electrochemistry to pharmaceuticals and high-tech composite materials. Among the many methods for producing nanoparticles, those that use renewable plant biomass or its extracts, as well as biogenic approaches for synthesizing nanoparticles within living cells, are particularly promising from the viewpoint of Green Chemistry and sustainable development. These techniques, which are part of the rapidly growing field of Nanobiotechnology, can help solve problems associated with the use of toxic or expensive chemicals and increase the sustainability and affordability of the production of nanoparticles and biohybrid materials based on them. This review explores various methods for creating nanoparticles from both precious and base metals, using a variety of reducing agents and enzymes found in plants and bacteria, as well as promising biochemical approaches involving the reduction of metal salts inside living cells.

## 1. Introduction

**Figure 1:**
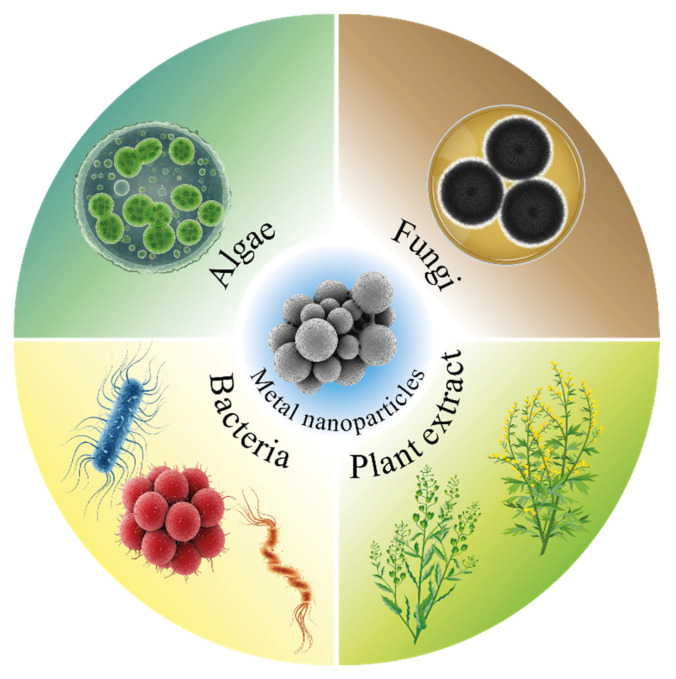
Biological materials used in the production of nanoparticles.

The intensive development of nanotechnology in recent decades has had a significant impact on various fields of science and technology, including in the field of obtaining new materials [1]. In recent years, metal nanoparticles have become widespread among nanomaterials due to the possibility of their use in fields such as catalysis, environmental remediation, electronics manufacturing, and many others [2,3]. The unusual properties of nanoparticles are related primarily to their small size (1–100 nm) and significantly larger surface area, which results in the high reactivity of such materials [4]. Moreover, their chemical reactivity can be controlled by changing their size, shape, and dispersion. Metal nanoparticles can be synthesized via various methods, including physical, chemical and biological methods. Physical methods are approaches in which temperature or radiation treatment of initial salts or oxides is used to generate nanoparticles [5,6,7], whereas in chemical synthesis methods, the initial materials react to form metallic nanoparticles in the presence of reducing agents such as sodium borohydride (NaBH_4_), hydrazine, and others. Importantly, these reducing agents are usually toxic in nature, which causes human health problems and pollutes the environment [8]. Therefore, to solve these problems, methods for the biosynthesis of nanoparticles using plants and their extracts, bacteria and other various biological materials have been proposed since these methods are simpler, biocompatible, less toxic, more energy efficient and environmentally friendly. These methods can be divided into several types: for example, the production of nanoparticles using microbial cells allows the synthesis of metal nanoparticles both on the surface and inside cells, and such compositions can serve as heterogeneous catalysts that are effective analogs to catalysts on carbon substrates. The synthesis of nanoparticles using a cell-free extract of microorganisms, fungi or yeast (a nutrient medium containing substances released by organisms during their growth) makes it possible to obtain stabilized colloidal systems containing metal nanoparticles (Figure 1) that can later be used in various fields, such as photocatalysis, the creation of microbial fuel cells or the production of antibacterial materials, and drugs [9,10]. The presented review compares favorably with modern works in its complexity and depth of analysis. Unlike most reviews, which are limited to 2–4 metals and general synthesis schemes, the presented work systematically analyzes approaches to the synthesis of nanoparticles based on eight metals with details of the proposed mechanisms of synthesis of nanoparticles, which is devoted to a separate subsection of the review [9,11,12]. The main drawback of existing reviews is their fragmented approach: they often focus either on synthesis methods or on practical applications separately but rarely integrate both aspects. Many works demonstrate narrow specialization, considering only one or two metals (mainly silver and gold) or limiting themselves to a single source of biosynthesis, such as plant extracts or algae. The paper also provides extensive systematic tables highlighting the most important characteristics of synthesized nanoparticles and areas of their further application [13,14,15,16].

In this review, methods for obtaining nanoparticles of precious and base metals from various living organisms and their metabolites, as well as applications of the obtained materials and biocomposites, are considered. Special attention has been given to the production of nanoparticles via microorganisms and the mechanisms by which this is possible.

## 2. Mechanisms for the Production of Metal Nanoparticles on Living Substrates

### 2.1. Mechanisms for the Production of Metal Nanoparticles via Bacteria

**Figure 2:**
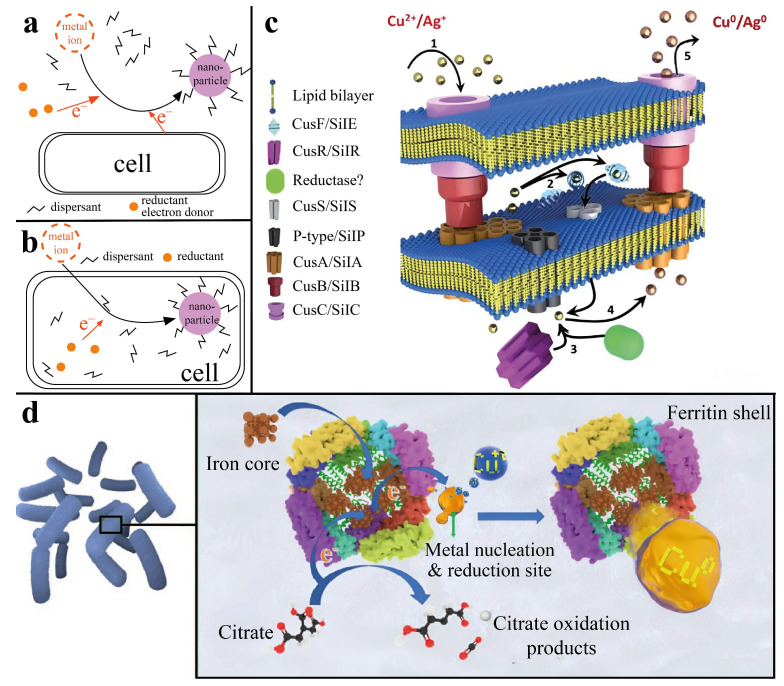
Mechanisms of the formation of CuNPs by microorganisms: (**a**) general view of the mechanism of synthesis of metallic NPs outside cells due to the use of external reducing agents [17] © 2020 by the authors; (**b**) general view of the mechanism of synthesis of metallic NPs inside cells due to the participation of redox enzymes in the cytoplasm [17] © 2020 by the authors; (**c**) proposed mechanism of reduction of CuNPs on the basis of the resistance of the microorganism *M. morganii* to AgNPs [18] © The Royal Society of Chemistry 2013; (**d**) proposed mechanism of synthesis of CuNPs owing to the participation of protein ferritin [19] © 2021 The Authors.

The production of metal nanoparticles via microorganisms is possible via two mechanisms. First, metal nanoparticles are obtained during the interaction of precursors with cell metabolites in the culture medium or with other reducing agents, resulting in the extracellular formation of nanoparticles. The second approach involves the penetration of a metal precursor into the cell of a microorganism, resulting in the reduction of nanoparticles as a result of interaction with enzymatic complexes (Figure 2a,b) [17].

The mechanisms of the intracellular production of metal nanoparticles seem to be the most interesting, since synthesis occurs without the use of various chemical reducing agents and allows cells to act as carriers or substrates for nanoparticles. Therefore, on the basis of the mechanism of resistance of the microorganism *Morganella morganii* RP42 to silver, a mechanism for the formation of metallic copper nanoparticles without the use of external reducing agents is proposed. This process consists of the absorption of Cu^2+^ ions by the bacterium, followed by the transfer of ions to the enzyme complex of the cell, which triggers a cascade of enzymatic reactions during which either a metal ion reductase or similar proteins can bind to Cu^2+^ ions, thereby reducing them to Cu^0^ nanoparticles, after which the nanoparticles are released from the cell via deductions in the cellular system. The size of the obtained nanoparticles ranged from 15 to 20 nm (Figure 2c) [18].

Another method of producing metal nanoparticles is associated with the presence of iron–containing proteins, ferritins. With the help of bacteria of the genus *Bacillus*, it was possible to obtain copper nanoparticles intracellularly by cultivating microorganisms in a solution of copper sulfate for 48 h. The proposed mechanism for the synthesis of nanoparticles consists of the reduction of copper during its interaction with ferritins capable of interacting with metal ions and restoring them. This process occurs via the transfer of electrons during the oxidation of citrate, which is located in the electron transfer chain and is part of a complex enzymatic mechanism in microbial cells that is responsible for the oxidation of glucose and the production of energy in the body (Figure 2d). The average size of the obtained nanoparticles was approximately 1.9 nm, which was determined via TEM [19].

**Figure 3:**
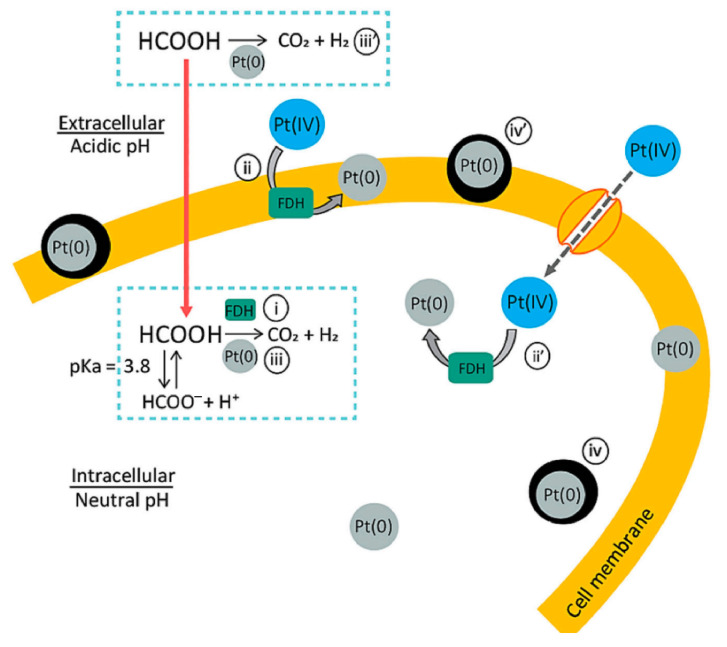
Putative mechanism of the intracellular formation of PtNPs [20] © 2021 by the authors.

Another mechanism for the production of metal nanoparticles in acidophilic bacteria consists of the reduction of nanoparticles in an acidic environment due to the decomposition of formic acid (FA) under the action of formate dehydrogenase (FDG) to form carbon dioxide and hydrogen, which act as reducing agents in the production of nanoparticles. Similarly, platinum nanoparticles were obtained from two strains of acidophilic bacteria: *Acidocella aromatica* and *Acidiphilium crytpum* [20]. Owing to the ability of FA to penetrate through the cell membrane into the periplasmic space of the cell, platinum nanoparticles (PtNPs), which act as chemical catalysts for the decomposition of the leaked FA, were obtained. This autocatalytic reaction led to the growth of the PtNP crystals, which stopped growing because of a decrease in the number of crystallization centers due to the inactivation of the FDG containing Cu^2+^ in the active center (Figure 3).

**Figure 4:**
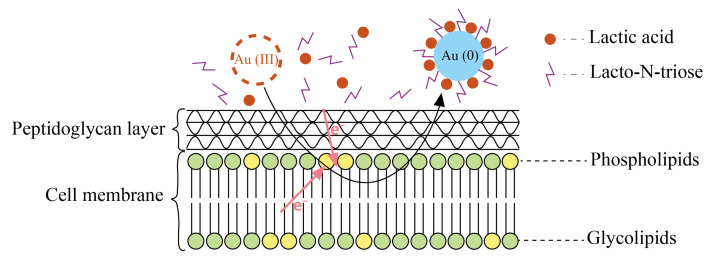
The mechanism of synthesis of gold nanoparticles is mediated by the components of the cell membrane [21] © 2019 Wiley-VCH Verlag GmbH & Co. KGaA, Weinheim.

A similar mechanism, accompanied by the penetration of metal ions into the cell membrane and the subsequent reduction of nanoparticles, was observed in the production of gold nanoparticles. In this case, the synthesis was due to the presence of diglycosyldiacylglycerols (DDGs) in the cell membrane of *Lactobacillus casei*, which, together with lacto-*N*-triose, act as electron donors for gold ions. The resulting nanoparticles were additionally stabilized on the surface of the outer membrane under the action of lactic acid (Figure 4) [21].

**Figure 5:**
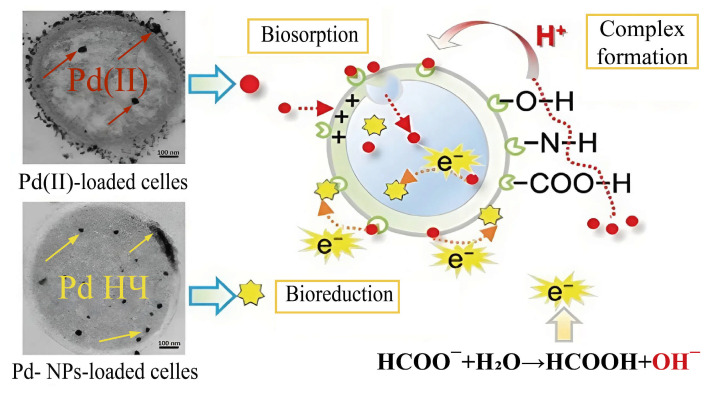
The mechanism of PdNP synthesis based on the processes of biosorption and bio-repair [22] © 2017 Elsevier B.V.

In the extracellular synthesis of metal nanoparticles, substances formed during the cultivation of microorganisms or functional groups located on the surface of the cell wall often play an important role. Because of biosorption, due to the presence of amino, carboxyl and hydroxyl groups, Pd^2+^ ions are fixed on the cell wall of *Enterococcus faecalis* microorganisms; in addition, some of the ions penetrate into the cell. The addition of formate as an electron donor initiated the reduction of palladium ions, which formed PdNPs during further aggregation (Figure 5) [22].

Intracellular copper nanoparticles were also obtained via the bacteria *Shewanella oneidensis*. The size of the synthesized nanoparticles varied from 20 to 50 nm, and the presence of various clusters was recorded. When examined via TEM, Cu_2_O nanoparticles with a size of less than 10 nm were also detected, which were copper nanoparticles coated with a thin oxide film formed during storage in air. The obtained nanoparticles were used as catalysts for the azide–alkyne cycloaddition reaction via the interaction of benzyl azide with 3 different terminal alkynes, and depending on the alkyne, the reaction yield ranged from 51% to 79% [23].

**Figure 6:**
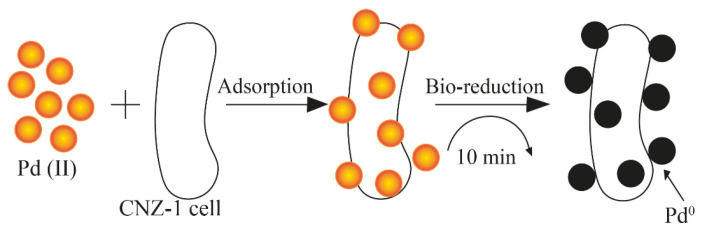
Scheme of the synthesis of PdNPs mediated by the presence of hydrogenases [24] © The Royal Society of Chemistry 2017.

The mechanism of PdNP synthesis using *Shewanella* sp. CNZ-1 was based on the sorption of palladium ions and their subsequent reduction due to an electron donor, sodium lactate, and hydrogenases, whose important role in the bioreduction process was proven by the inhibition of enzyme activity when a Cu^2+^ salt solution was added (Figure 6) [24].

**Figure 7:**
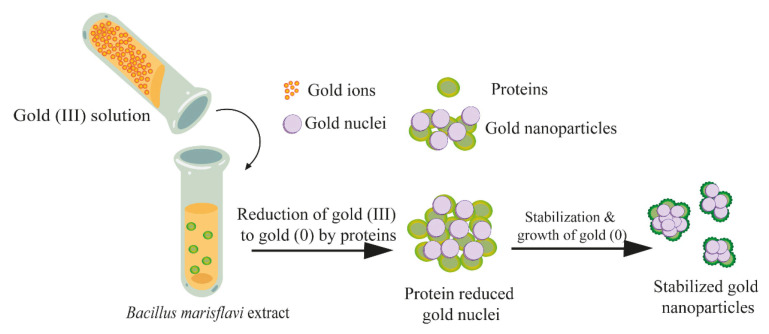
The mechanism of the formation of gold nanoparticles mediated by proteins in the cell-free extract of *B. marisflavi* [25] © 2016 The Authors.

In addition to the participation of microorganisms themselves in the synthesis of nanoparticles, substances formed as a result of the cultivation of microorganisms can participate in the reduction process. Thus, the proteins contained in the cell-free extract of the *Bacillus marisflavi* culture acted as reducing agents and stabilizers of the gold nanoparticles, reducing their degree of agglomeration (Figure 7) [25].

Thus, microorganisms represent unique platforms for the efficient and environmentally friendly synthesis of nanoparticles of various metals, combining biocompatibility and versatility. Further research should focus on process optimization, the study of new strains, and the integration of biogenic nanomaterials into practical applications.

### 2.2. Mechanisms for the Production of Metal Nanoparticles via Plant Extracts

The production of metal nanoparticles via plant extracts is similar in mechanism to the production of nanoparticles via a microbial supernatant because plants also contain many substances capable of acting as reducing agents in this process.

**Figure 8:**
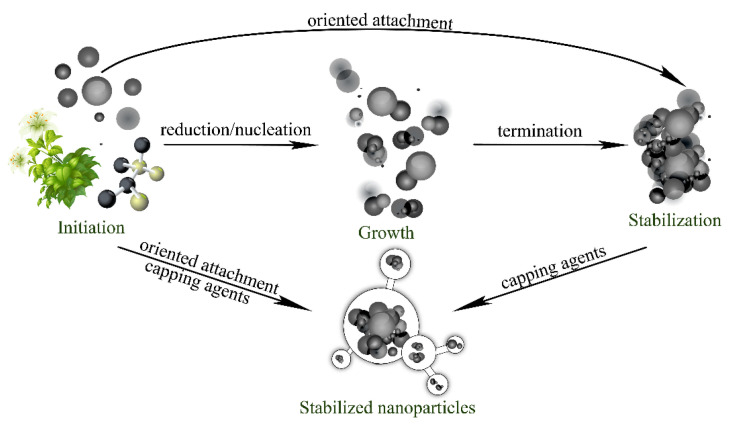
Stages of nanoparticle formation.

Plant-mediated nanoparticle synthesis proceeds through four distinct sequential phases, each governed by specific chemical processes (Figure 8). The initiation phase encompasses metal ion reduction and primary nucleation events. During this phase, reduced metal atoms (M^0^) accumulate to critical concentrations, overcoming nucleation barriers and forming primary clusters containing 40–100 atoms. The subsequent growth phase involves aggregation and coalescence of primary particles into larger secondary structures. Particle coalescence proceeds through oriented attachment mechanisms, wherein particles align along specific crystallographic planes before fusing, a process driven by surface energy minimization and thermodynamic stability considerations. The stabilization phase involves preferential adsorption of capping agents onto high-energy crystal surfaces. This process inhibits the growth of energetically unfavorable crystallographic planes while promoting the development of specific morphologies. The termination phase finalizes the particle size and shape distributions, which are typically completed within. For *Azadirachta indica* (neem) extract synthesis, maximum silver nanoparticle production occurs after 2 h of reaction time, yielding particles 5–35 nm in diameter with optimal absorption rates [26].

**Figure 9:**
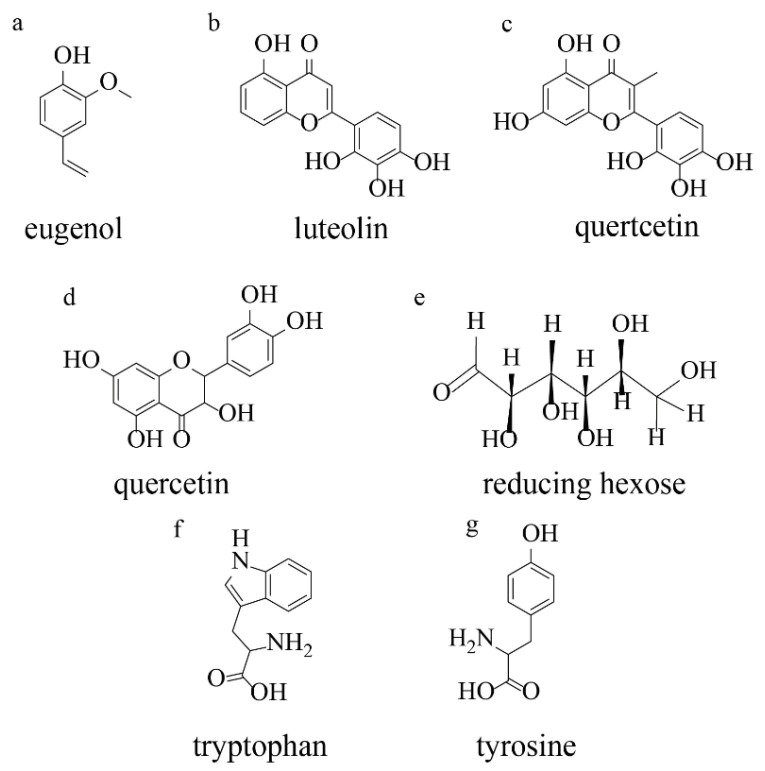
Metabolites involved in the production of nanoparticles: (**a**) terpenoids (eugenol); (**b**) flavonoids (luteolin); (**c**) flavonoids (quertcetin); (**d**) flavonoids (quercetin); (**e**) a reducing hexose with the open chain form; (**f**) tryptophan; (**g**) tyrosine. Adopted from [26] © 2014 Park-media, Ltd.

Plant extracts represent complex mixtures of secondary metabolites that facilitate metal nanoparticle formation through coordinated reduction and stabilization processes (Figure 9). The primary reducing agents identified include flavonoids, phenolic acids, terpenoids, and proteins, each contributing distinct reduction mechanisms. Among these compounds, flavonoids demonstrate exceptional reducing capacity through enol-to-keto tautomerization. When flavonoid molecules undergo tautomeric conversion from the enol form to the keto form, reactive hydrogen atoms are released, enabling effective electron transfer to metal ions. For example, quercetin, a representative flavonoid, contains three distinct chelation sites—the C3-hydroxyl, C5-hydroxyl, and C3′-C4′-catechol groups—capable of reducing up to two moles of Ag^+^ or Au^3+^ ions per quercetin molecule.

Phenolic acids, particularly chlorogenic acid and caffeic acid, also serve as effective bioreductants through hydrogen-donating mechanisms. The hydroxyl-rich structure of phenolics enables sequential one-electron transfer to metal ions, progressively reducing Au^3+^ to Au^0^ or Ag^+^ to Ag^0^. Analysis of post-reaction residues through ultra-performance liquid chromatography demonstrated substantial consumption of phenolic compounds during synthesis, with concentrations decreasing from micromolar to nanomolar levels. Terpenoid compounds, such as eugenol found in cinnamon extracts, contribute to metal reduction through deprotonation of their hydroxyl groups, yielding nucleophilic anions capable of transferring electrons to metal centers.

Complementing the reduction phase, plant extracts contain distinct classes of stabilizing biomolecules that adsorb onto nascent nanoparticle surfaces, preventing aggregation and controlling the final particle dimensions. Xanthones, phloroglucinols, and other low-polarity compounds accumulate on nanoparticle surfaces, with FTIR analysis confirming their presence through characteristic functional groups, including methoxy and prenyl moieties. The common structural feature distinguishing reducing agents from stabilizing agents is the presence of an enol group in reductants versus methoxy and/or prenyl groups in capping agents. When compounds possess both functional group classes, they perform dual reduction-capping functions, exemplified by naphthodianthrones in plant extracts [27,28,29,30].

**Figure 10:**
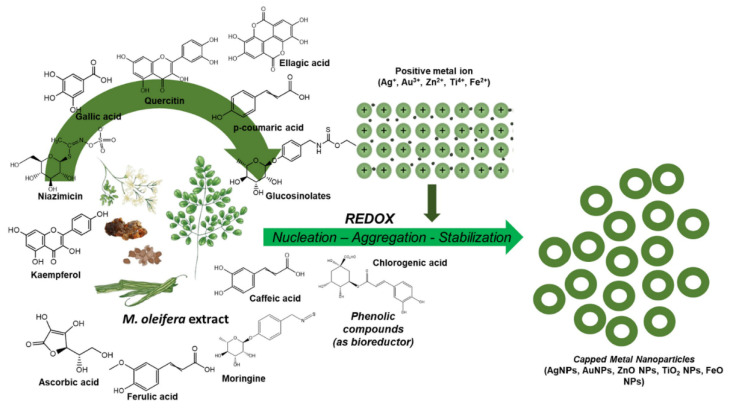
Mechanism of synthesis of metal nanoparticles in the presence of polyphenolic compounds, flavonoids and terpenoids [31] © 2024, The Authors.

Polyphenolic compounds, flavonoids, and terpenoids contained in the extract of the *Moringa oleifera* plant actively interact with metal ions, resulting in the formation of reduced forms of metals that serve as centers for further crystallization. The functional groups of secondary metabolites (hydroxyl -OH, carboxyl -COOH, and amine -NH_2_) are adsorbed on the surface of the nanoparticles, forming a protective layer. This prevents them from sticking together and ensures stability. For example, the polysaccharides and proteins in the extract act as anticoagulants, fixing the shape and size of the NPs (Figure 10) [31].

**Figure 11:**
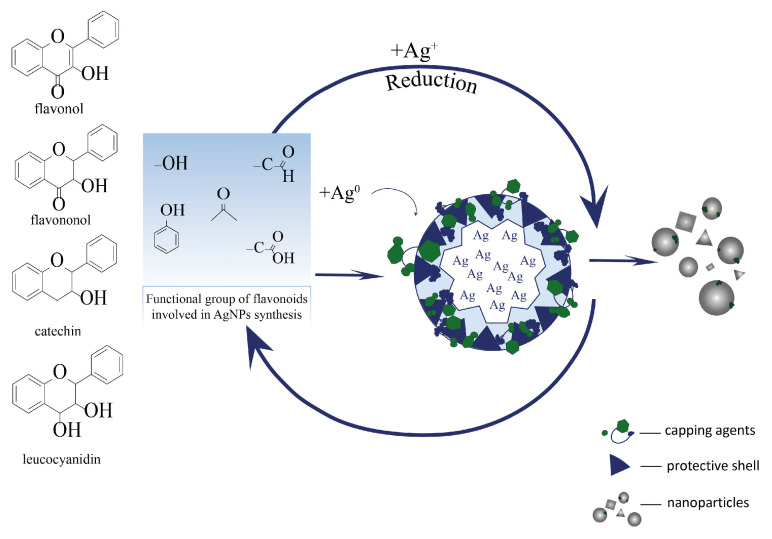
The process of stabilization of silver nanoparticles by flavonoids.

Flavonoids play a complex and multistep role in the recovery and stabilization of silver nanoparticles when plant extracts are used. Owing to the presence of many hydroxyl groups in their structure, especially in ortho-positions in the aromatic ring, these groups are ionized (deprotonated) in an aqueous medium, which allows flavonoids to donate electrons to reduce silver ions Ag^+^ to the metallic state Ag^0^ (Figure 11) [32].

## 3. Strategies for Size and Morphology Control

The process of biogenic formation of nanoparticles is influenced by many factors in addition to the nature of the reducing agent, such as temperature, optimal pH, and the presence or absence of substances that prevent the agglomeration of nanoparticles.

The most important factor among the above factors is temperature, the change in which can critically affect the size of the formed nanoparticles, as shown by the example of AgNPs obtained using *Bacillus cereus* microorganisms. When the temperature changed from 25 °C to 50 °C, the synthesis of nanoparticles by microorganisms accelerated, and their size decreased to an average of 5–7 nm. This was also associated with the influence of pH: at an optimum pH of 9, the enzymatic systems responsible for the reduction of silver ions were activated, which made it possible to obtain particles after 69 h [33].

Earlier studies also investigated the effects of temperature and pH on AgNPs biosynthesis over wide ranges: from 0 °C to 100 °C and from 3 to 11 pH. The *Fusarium oxysporum* filtrate used for the synthesis of AgNPs made it possible to achieve the optimal size of nanoparticles in the temperature range of 40–60 °C and an alkaline pH. Lowering the temperature below 40 °C led to inactivation of enzymes and a reduction in metabolites and, as a result, deterioration in the production of nanoparticles, and an increase in the temperature above 60 °C reduced the stability of the colloidal system due to the denaturation of stabilizing proteins. Similar to an increase in temperature, a pH shift to the acidic region led to increased aggregation of the nanoparticles due to a change in the charge of the proteins that stabilize the nanoparticles [34]. Subsequent studies have shown that in addition to the optimum pH and temperature, it is necessary to observe the concentration of the precursor of the nanoparticles. Thus, the addition of 2 mmol of AgNO_3_ solution to the filtrate of the same *F. oxysporum* culture made it possible to obtain the optimal AgNPs in size. By analogy with early studies, a decrease in the concentration of the precursor worsened the production of nanoparticles, and exceeding this value contributed to cluster formation owing to the adhesion of unstable particles [35].

The rate of nanoparticle production also depends on an increase in temperature, as demonstrated by a study of 29 thermophilic fungi that demonstrated the best production of gold nanoparticles at 45 °C. The influence of temperature made it possible to achieve optimal fermentation in mushrooms and accelerate synthesis for up to 20 h. Researchers have also studied various forms of the supernatant from the selected fungi: filtrates containing metabolites, autolysate, and intracellular extracts. The use of each of these supernatants made it possible to obtain AuNPs of the same size; however, the use of the filtrate made it possible to obtain stable nanoparticles due to the presence of substances with masses greater than 3 kDa [36]. A similar approach, which involves selecting a reducing agent or its form in cases analogous to the one described above, allows control over the size and morphology of nanoparticles alongside changes in synthesis conditions. One such method is controlled intracellular or extracellular nanoparticle formation. In a similar manner, the use of *Bacillus cereus* bacteria has led to the synthesis of predominantly spherical AgNPs both intracellularly and extracellularly. The greater number of binding agents (peptidoglycan present in the cell wall) caused some nanoparticles to change their shape to ellipsoidal and doubled their average size [37]. In contrast, employing the same microorganism for the intracellular synthesis of CuNPs yielded uniformly distributed particles smaller than 1.9 nm at room temperature within 48 h owing to interactions with iron-containing proteins—ferritins [19].

Additionally, nanoparticle size can be controlled by varying the biological source of the reducing agent. Bacterial systems enable precise regulation of synthesis conditions to produce either intracellular or extracellular nanoparticles due to the presence of diverse enzymatic systems [18,19,21,22] as well as the metal-specific affinity of certain microorganisms [20]. Plant extracts, in turn, yield diverse nanoparticle morphologies due to the broad array of reductants present (flavonoids, phenolic and terpenoid compounds), which also serve as capping agents (xanthone and phloroglucinol compounds), enabling the production of cubic, spherical, and polygonal nanoparticles [38,39,40]. Certain fungal cultures have attracted significant interest because of their ability to produce small, dispersed nanoparticles [41]. In contrast, other fungi tend to form agglomerated particles owing to the limited production of stabilizing proteins or analogous compounds [42,43].

These differences underscore the need to account for numerous factors influencing nanoparticle synthesis via biological materials, and modern machine learning methods assist in modeling experiments and optimizing multiple parameters [33].

## 4. Nanoparticles Produced via Bacteria

In this section, various methods for the production of metal nanoparticles via microorganisms and their metabolites in culture media are considered. The green synthesis of nanoparticles using microorganisms is an environmentally friendly approach that uses renewable materials to restore metals and stabilize nanoparticles. This method eliminates the formation of toxic chemical byproducts and is characterized by the low toxicity of finished nanomaterials. This process is carried out because biologically active metabolites and enzymatic systems are present in microbial cells. Biosynthetic processes can be implemented by both intracellular and extracellular mechanisms, which provides flexibility in the choice of methods for the synthesis of biogenic nanoparticles (Table 1) [44,45,46].

The table shows the versatility of bacterial systems in synthesizing nanoparticles from virtually all relevant metals and their oxides. Microorganisms synthesize nanoparticles of diverse compositions, from precious metals (gold, silver, platinum, and palladium) to more common and accessible ones (copper, nickel, iron, zinc, and cobalt). Compared with extracellular nanoparticles, intracellular nanoparticles are smaller and narrower in size because of their complex enzymatic systems and bacterial protective mechanisms. Biosynthesized nanoparticles predominantly serve as antibacterial agents (Ag, Cu, and CuO), whereas many bacteria produce catalytically active nanoparticles for cross-coupling reactions (Pt, Pd, and Cu), *p*-nitrophenol and Cr(VI) reduction (Pd and Cu), and dye decolorization (Cu).

### 4.1. Au Nanoparticles

Since ancient times, colloidal solutions of gold nanoparticles have been among the most stable forms of all metal nanoparticles [77]. These nanoparticles are used as catalysts for organic synthesis; in medical applications, including chemotherapy and drug delivery; and in electrochemistry and other fields [78,79,80,81].

Numerous studies have confirmed that various microorganisms have a unique ability to biosynthesize metal nanoparticles. In particular, *Escherichia coli* bacteria, which are classic model systems for biological research, have demonstrated a pronounced ability to form gold nanoparticles (AuNPs). Studies have shown that synthesized particles approximately 50 nm in size are predominantly localized in the cell membrane. Importantly, these nanoparticles showed high catalytic activity for the reduction of 4-nitrophenol in the presence of NaBH_4_. Importantly, the functional groups of the *E. coli* cell wall effectively stabilize the nanoparticles, which makes these microorganisms promising for creating “green” catalytic systems [82].

With respect to thermophilic microorganisms, the bacteria *Caldicellulosiruptor changbaiensis* should be highlighted. Owing to their ability to produce hydrogen during growth, these unique microorganisms have demonstrated the possibility of synthesizing not only gold (AuNPs) but also palladium (PdNPs) nanoparticles. Notably, the obtained nanoparticles were exceptionally small (1.65 nm for the AuNPs and 1.61 nm for the PdNPs) and had a spherical morphology. Studies of their catalytic activity have shown impressive results: AuNPs provide complete reduction of o-nitroaniline in just 10 min, whereas PdNPs demonstrate 100% conversion in the reaction of asymmetric isoforone hydrogenation, significantly exceeding the efficiency of the traditional Pd/C catalyst [83].

**Figure 12:**
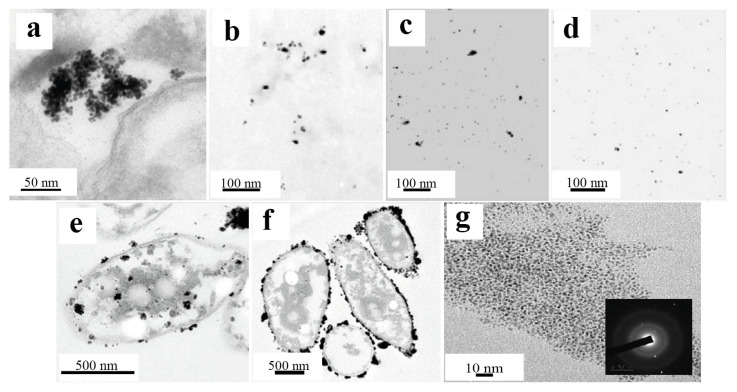
Morphology and size of the NPs produced from the precious metals: (**a**) TEM image of the AuNPs on an ultrathin section of *R. sphaeroides* cells treated with a 10 µm Au(III) solution [84] © 2018 Elsevier B.V.; (**b**) TEM image of the AgNPs synthesized from *P. antarctica* culture supernatants [47] Copyright © 2011 Elsevier Ltd., Amsterdam, The Netherlands; (**c**) TEM image of the AgNPs synthesized from *A. kerguelensis* culture supernatants [47] Copyright © 2011 Elsevier Ltd., Amsterdam, The Netherlands; (**d**) TEM image of the AgNPs synthesized from the culture supernatants of *A. gangotriensis* [47] Copyright © 2011 Elsevier Ltd., Amsterdam, The Netherlands; (**e**) TEM image of the PtNPs produced from active *Ac. crytpum* cells, using 10 mM formate [20] © 2021 by the authors; (**f**) TEM image of PtNPs obtained by active *Ac. aromatica* cells using 20 mM formate [20] © 2021 by the authors; (**g**) TEM image of PtNPs obtained from *P. stutzeri* (the inset shows an image of the selected region with an electron diffraction pattern) [85] © 2020 Elsevier Ltd., Amsterdam, The Netherlands.

In continuation of the analysis of biosynthetic approaches, the results obtained with the phototrophic bacteria *Rhodobacter sphaeroides* are of significant interest. During a 10-day incubation with hydrogen tetrachloroaurate, these microorganisms formed well-dispersed spherical gold nanoparticles measuring 5–18 nm (average size of 10 nm), nevertheless forming rather large clusters (approximately 100 nm) (Figure 12a). Notably, despite the larger size compared with the particles synthesized by *C. changbaiensis*, the AuNPs data also revealed pronounced catalytic activity in the 4-nitrophenol reduction reaction [84].

### 4.2. Ag Nanoparticles

Silver nanoparticles have become very widespread due to their potential for use in various fields, such as processing and creating medical instruments, delivering medicines, creating electronics, and many others [86,87]. Importantly, the antibacterial properties of silver ions during the production of AgNPs are important. Extracellular bacterial substances, which can also constitute a matrix of microorganisms, are responsible for the protective reactions and resistance of bacteria. These substances can act as reducing agents, thereby deactivating some of the ions and turning them into nanoparticles, which pose less danger to microorganisms than free silver ions do. Thus, in the synthesis process, to avoid cell death, it is necessary to calculate the minimum inhibitory concentration strictly and not exceed its value for the effective production of nanoparticles [88].

Studies of the antibacterial activity of silver nanoparticles (AgNPs) obtained from a cell-free extract of five strains of psychrophilic bacteria (*P. antarctica*, *P. proteolytica*, *P. meridiana*, *A. kerguelensis* and *A. gangotriensis*) and two mesophilic microorganisms of the genus *Bacillus* (*B. indicus* and *B. cecembensis*) have demonstrated their pronounced antimicrobial activity. Experiments have shown that a solution of silver nanoparticles at a concentration of 2 mg/mL effectively suppresses the growth of four cultures, which does not interfere with the synthesis of nanoparticles: *P. antarctica*, *A. kerguelensis*, *A. gangotriensis* and *B. indicus*, whereas a higher concentration of 10 mg/mL is required to suppress the growth of *E. coli* and *P. proteolytica*. Compared with *P. antarctica* (Figure 12b) and *A. kerguelensis* (Figure 12c), *A. gangotriensis* (Figure 12d) synthesized the smallest and better distributed particles. Notably, the size of the synthesized nanoparticles varied significantly depending on the producing microorganism, from 2.2 nm to 33.6 nm [47].

The results of the use of biogenic silver nanoparticles synthesized with *Bacillus subtilis* to combat infectious diseases are particularly interesting. Despite the use of a cell-free extract, which often leads to particle agglomeration, well-dispersed AgNPs ranging in size from 3 to 20 nm were obtained in this case, which was confirmed by TEM. Studies have shown that at a concentration of 200 micrograms/mL, compared with standard antibiotics, biosynthesized silver nanoparticles are more effective against microbial pathogens and fungi. Quantitative assessment of AgNP activity, which is based on indicators of the minimum inhibitory concentration (MIC) and minimum lethal concentration (MLC), revealed the following ranges: MIC—50–205 mcg/mL and MLC—220–500 mcg/mL [48].

Further studies of the antimicrobial properties of AgNPs synthesized from a cell-free extract of the cyanobacteria *Anabaena variabilis* revealed an interesting synergistic effect when nanoparticles were combined with standard antibiotics (streptomycin, amphotericin B and fluconazole). Experiments with pathogenic microorganisms (*E. coli*, *K. pneumoniae*, *B. cereus*, and *P. aeruginosa*) have shown that the combined use of nanoparticles and antibiotics can significantly (several times) reduce the required concentrations of both components against both bacterial pathogens and fungi [49].

### 4.3. Pt Nanoparticles

Platinum nanoparticles enhance the effectiveness of chemotherapy by generating reactive oxygen species and targeting tumor cells while simultaneously demonstrating peroxidase and catalase activity to regulate redox homeostasis. In the energy sector, PtNPs serve as highly efficient electrocatalysts for the reaction of hydrogen and fuel cell release, and their green biosynthesis from plant extracts enables the production of biocompatible catalysts with antibacterial properties [89,90].

A promising approach in the biosynthesis of nanoparticles is the use of biofilms, which has been successfully demonstrated by the example of the microorganism *Shewanella loihica*. In this study, after biofilm formation, gold (AuCl_3_) and palladium (PdCl_2_) chlorides, as well as chloroplatinic acid (H_2_PtCl_6_), were added to the medium, which made it possible to obtain nanoparticles of three metals simultaneously for 2 days with constant stirring. All synthesized nanoparticles had a spherical shape, and their size was dependent on the pH of the medium: at pH = 7, the sizes for Au were 2–7 nm, those for Pt were 2–6 nm, and those for Pd were 2–6 nm, whereas at pH = 9, an increase in size was observed to be 2–10 nm for Au, 2–10 nm for Pt and 2–12 nm for Pd. Special attention should be given to the high catalytic activity of the obtained palladium nanoparticles, which ensures the complete decomposition of the methylene orange dye in just 3 min, as evidenced by the complete discoloration of the reaction mixture [91].

An alternative system for the synthesis of metallic nanoparticles is acidophilic bacteria, whose specific metabolic pathways have been used to produce catalytically active platinum nanoparticles. The study used two strains of such bacteria, *Acidocella aromatica* and *Acidiphilium cryptum*. Interestingly, although the nanoparticles obtained in both cases retained a spherical shape, their average sizes differed significantly: 16.1 nm for *Acidocella aromatica* and 28.9 nm for *Acidiphilium cryptum*. At the same time, large clusters of nanoparticles were observed on the cell wall and inside the cells themselves (Figure 12e,f), which may be due to the small amount of stabilizing substances. The greatest tendency toward agglomeration was shown by *Acidiphilium cryptum*. The catalytic activity of these nanoparticles was demonstrated in the reduction of Cr(VI) to Cr(III), where depending on the composition of the microorganism/PtNPs system, 1 mg of the composition accounted for between 1.3 and 3.3 mg of reduced Cr(VI). Notably, this efficiency exceeded the performance of the model Pt/C catalyst, which highlights the promise of the use of biogenic nanoparticles [20].

Of particular interest are the results obtained via *Pseudomonas stutzeri*, which demonstrated the ability to synthesize both PdNPs and PtNPs. All the obtained particles retained a spherical morphology, while the sizes ranged from 2 to 5 nm for platinum and from 5 to 10 nm for palladium. Catalytic tests revealed that PdNPs effectively catalyzed the reduction of 4-nitrophenol to 4-aminophenol, achieving 60% conversion in 5 min and 92% conversion in 30 min. At the same time, synthesized platinum nanoparticles (Figure 12g), which are well distributed and do not form large clusters, demonstrated pronounced antioxidant activity and the ability to suppress the hemolysis process, which opens up prospects for their use in medicine [85].

### 4.4. Pd Nanoparticles

Palladium has been applied as an industrial catalyst with paramount applications, where owing to its high cost, it is mainly used in the form of nanoparticles stabilized on an inert substrate, which makes it possible to achieve high catalysis efficiency and stability over many catalytic cycles [92,93].

**Figure 13:**
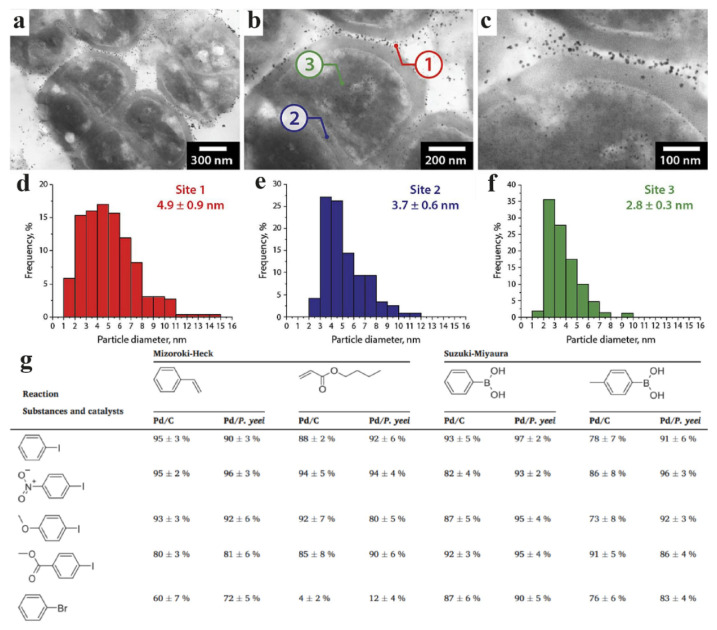
Catalyst based on PdNPs and *Paracoccus yeei*: (**a**) image of the catalyst at ×50 k magnification; (**b**) ×100 k magnification; (**c**) ×200 k magnification; (**d**) distribution of palladium nanoparticles on the cell surface; (**e**) between individual cells in sarcines; (**f**) inside cells; (**g**) substrates and yields of products of catalyzed reactions [62] © 2023 Elsevier Inc.

Biohybrid preparations based on cells of various bacteria acting as substrates for catalytically active palladium nanoparticles have opened a new direction in heterogeneous catalysis. Thus, PdNPs obtained from *Paracoccus yeei* were highly efficient in classical cross-coupling reactions and were not inferior or even superior to a commercially available carbon-based catalyst. In addition, the resulting biohybrid catalyst maintained its effectiveness for 5 consecutive reaction cycles (Figure 13) [62]. In the development of the study, the authors developed a fast method for synthesizing palladium nanoparticles, which requires only 7 min. The resulting biohybrid catalyst demonstrated high activity and selectivity in organic synthesis, surpassing its commercial counterpart, Pd/C, and the nanoparticles located on the surface of the cell wall and inside the cells themselves did not undergo agglomeration owing to the presence of enzymes [63].

**Figure 14:**
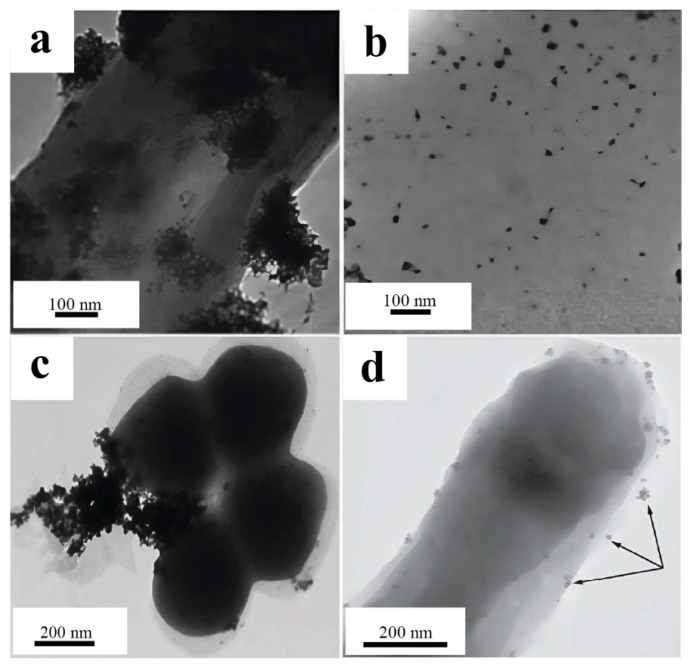
Comparison of PdNP images obtained from microorganisms: (**a**) PdNPs obtained with *P. putida*; (**b**) PdNPs obtained with *C. necator* [66] © The Royal Society of Chemistry 2009; (**c**) PdNPs obtained with *E. faecalis* [65] Copyright © 2015 Elsevier B.V.; (**d**) PdNPs obtained with *C. metallidurans* CH34 and stabilized in glutaraldehyde [64] © 2020 Elsevier B.V.

Palladium nanoparticles localized in the periplasmic space of *Cupriavidus necator* and *Pseudomonas putida* microorganisms have been successfully used as biohybrid catalysts for cross-coupling reactions, including Suzuki–Miyaura and Mizoroki–Heck reactions. The catalyst based on *C. necator* has demonstrated excellent activity and high tolerance to functional groups in organic substrates, allowing effective reactions with 10 different aryl halides in the Suzuki–Miyaura reaction and 15 in the Mizoroki–Heck reaction. Moreover, the catalyst is based on *P. putida* and has been proven less versatile, as it catalyzes the Mizoroki–Heck reaction with only three substrates. The nanoparticles obtained with *Pseudomonas putida* (Figure 14a) formed large clusters of particles located on the surface of the cell wall, which significantly worsened the catalytic activity. The high activity of *C. necator* (Figure 14b) may be associated with a nanoparticle size not exceeding 10 nm, which provides a large specific surface area and accessibility of active sites [66].

Similar advantages of a small size (less than 10 nm) were revealed in the synthesis of palladium nanoparticles using *Enterococcus faecalis* (Figure 14c). The resulting nanoparticles were localized on the surface of the cell wall, forming large clusters larger than 500 nm and rare small clusters and single particles. However, owing to their thin cell wall, PdNPs are formed not only on the membrane but also inside the cells. These nanoparticles were used to catalytically reduce toxic Cr(VI) to Cr(III), demonstrating 42%, 98.9%, and 100% efficiency after 1, 6, and 12 h, respectively. Interestingly, *E. faecalis* cells themselves recovered up to 16% Cr(VI) without the addition of catalytically active nanoparticles, which highlights their natural reducing potential [65].

In another study, *Cupriavidus metallidurans* synthesized palladium nanoparticles localized on the cell wall. The key aspect of the work was the effect of stabilizing agents: aggregation of particles over 500 nm in size was observed in the formate medium, whereas the addition of glutaraldehyde allowed a stable colloidal solution with nanoparticles of 10–20 nm to be obtained (Figure 14d). The optimized PdNPs were used to reduce Cr(VI) and p-nitrophenol, achieving 92% and 90% efficiency in 110 and 330 min, respectively. This confirms that controlling the size and stability of nanoparticles is critically important for their catalytic activity [64].

### 4.5. Cu Nanoparticles

Copper nanoparticles are widely used in the field of medicine as antibacterial materials, but they have also been used as heterogeneous catalysts for organic synthesis reactions, particularly click reactions [94]. The production of such nanoparticles on a substrate from microorganisms is poorly understood and has only begun to develop in recent years, while much of the work has focused on using the resulting biohybrid materials as antiseptic agents rather than investigating their possible use in catalysis.

Importantly, sometimes because of the synthesis of nanoparticles, the death of microorganisms occurs; therefore, new methods of working with cell cultures in the synthesis of nanoparticles are being sought. For example, a cell-free extract obtained after separation of the microorganism *Serratia* sp. by centrifugation was used for the synthesis of copper nanoparticles. The particles obtained in this way were a mixture of Cu/CuO NPs with a predominant oxide content. In this case, intracellular copper nanoparticles were obtained, which resulted in the death of microorganisms and rupture of the cell wall; as a result, the copper nanoparticles entered the solution and, when they were oxidizing, were covered with an oxide film. The size of such particles ranges from 10 to 30 nm, and they are well dispersed [95].

**Figure 15:**
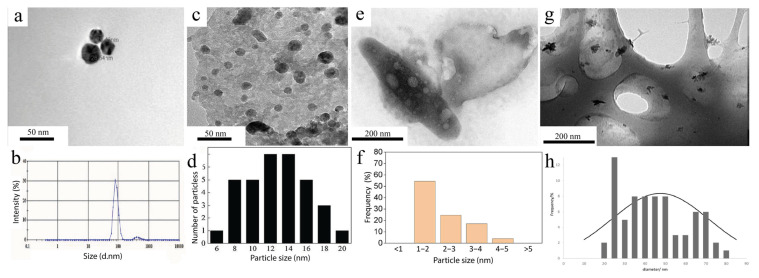
Comparison of CuNPs obtained with the help of opportunistic microorganisms: (**a**) TEM image of CuNPs synthesized by the strain *St. maltophilia* SCS1.1; (**b**) DLS analysis of the size of the copper nanoparticles synthesized by *St. maltophilia* SCS1.1 [50] © 2023 The Authors; (**c**) TEM image of the CuNPs obtained with *S. loihica*—PV4; (**d**) size distribution of the CuNPs obtained with *S. loihica*—PV4 [51] © 2017 Elsevier B.V.; (**e**) TEM image of the CuNPs in *P. stutzeri* biomass; (**f**) size distribution of the CuNPs in *P. stutzeri* biomass [72] © 2021 Elsevier Ltd., Amsterdam, The Netherlands; (**g**) TEM image of the CuNP samples obtained via *Rh. erythropolis* ATCC4277; (**h**) Size distribution of the CuNPs obtained via *Rh. erythropolis* ATCC4277 [98] © 2023 Elsevier Ltd., Amsterdam, The Netherlands.

Copper nanoparticles synthesized from a cell-free extract of the facultative anaerobic bacterium *Stenotrophomonas maltophilia* were obtained by culturing microorganisms with copper sulfate (CuSO_4_) for 48 h. The particles were spherical in shape, and their size ranged from 18.5 to 31.7 nm (Figure 15a,b). A study of their antibacterial activity revealed high efficacy against 14 phytopathogenic fungi and 9 pathogenic microorganisms. In addition, the nanoparticles demonstrated the ability to decompose pesticides: chlorpyrifos—by 90.4%; profenophos—by 87.2%; imidacloprid—by 51.3% [50].

In addition to *S. maltophilia*, another strain of the genus *Stenotrophomonas*, *S. rhizophilia*, was used to synthesize copper nanoparticles. NPs obtained from a cell-free extract after cultivation with copper ions were tested for their antimicrobial activity against *S. aureus*, *E. coli*, *P. aeruginosa*, *K. pneumoniae*, *S. typhi* and the fungus *C. albicans*. In addition to suppressing the growth of pathogens, the nanoparticles inhibited the formation of biofilms of *S. aureus* and *E. coli* by 89% and 97%, respectively, destroying the polysaccharide matrix and damaging cell membranes [96].

An alternative approach using *Shewanella loihica* made it possible to obtain spherical copper nanoparticles with a size of 6–20 nm (Figure 15c,d). During the synthesis process, nanoparticles are formed inside the cells of the microorganisms themselves, which can be realized via the mechanisms of soil bacteria and their mechanisms of adaptation to heavy metals [97]. Importantly, a decrease in the intracellular concentration of copper ions was observed, which was confirmed by linear scanning of the cell cross section. These nanoparticles effectively inactivate *E. coli*, which expands their use in the fight against bacterial infections [51].

Nanoscale copper particles were also obtained from *Kocuria flava* microorganisms isolated from the marine environment. The source in this case was not copper sulfate (CuSO_4_), which is most often used as a precursor during synthesis, but nitrate (Cu(NO_3_)_2_). The size of the nanoparticles obtained in this way varied from 10 to 30 nm, whereas their shape was spherical, as in the previously reviewed papers, but the authors of the work did not mention the presence of clusters, focusing on the first mention of the production of Si nanoparticles using microorganisms isolated from the marine environment [68].

Nanoparticles of copper and its oxides can also be obtained via culture of an isolate of actinomycete, the cell-free extract of which serves as a reducing agent for CuONP production. Crystal-shaped nanoparticles have been used to inhibit model human pathogens and microorganisms that have harmful effects on fish. The effectiveness of the obtained CuONPs was similar to that of the antibiotic ciprofloxacin against both human and fish pathogens [99].

To optimize methods of nanoparticle synthesis, native strains isolated from copper mines, as well as alternative sources of metal ions, can be used, which in turn opens the way for bioremediation and reduces the environmental burden. For example, *Bacillus cereus* microorganisms isolated from copper mines have been proposed for the synthesis of nanoparticles using copper sulfate (CuSO_4_) as a precursor. A comparison of native strains with laboratory strains revealed that natural strains adapted to copper form smaller nanoparticles: their size decreased from 26–97 nm to 11–33 nm. The obtained CuNPs demonstrated antimicrobial activity against the pathogens *E. coli*, *P. aeruginosa*, *B. subtilis* and *S. aureus*. Although their effectiveness was inferior to that of the antibiotic ciprofloxacin, the nanoparticles showed cytotoxicity against lung carcinoma cells, which opens up prospects for their use in cancer therapy [52].

A strain of *Pseudomonas stutzeri* was used to dispose of mining waste (“tailings” of copper ores). Nanoparticles synthesized by this microorganism tend to form agglomerates of nanoparticles 5–80 nm in size; however, detailed analysis revealed that individual particles have a diameter of 1–5 nm and are localized on the cell surface (Figure 15e,f). This method highlights the potential of biotechnology in the processing of industrial waste [72]. Unlike *P. stutzeri*, the *Rhodococcus erythropolis* ATCC4277 strain demonstrated greater efficiency in copper recovery from tailings. The resulting nanoparticles were 50–60 nm in size (the predominant size was 25 nm) and formed few clusters up to 80 nm in size (Figure 15g,h). However, the key limitation of this method is the need to use a cell-free extract isolated after 7–14 days of cultivation, since only by this time does the necessary concentration of cellular metabolites accumulate, which significantly slows the synthesis process. However, *Rh. erythropolis* ATCC4277 can be considered more promising for industrial applications because of its ability to control particle size [98].

The use of electronic waste, such as printed circuit boards, as a source of copper for the production of nanoparticles is also innovative and promising. In one study, a cell-free extract of the *Alcaligenes aquatilis* strain was used to restore copper ions from leached solutions obtained after processing boards with a mixture of hydrochloric and nitric acids. The synthesized copper oxide nanoparticles (CuO-NPs) were used to detoxify carcinogenic p-nitrophenol, achieving a 90% removal rate in 6 min. This approach not only solves the problem of electronic waste disposal but also creates the basis for “green” environmental cleaning technologies [67].

In addition to their antibacterial activity and catalysis, copper nanoparticles are used in a variety of related fields, such as agriculture, cytotoxic research, antitumor research, and various biotechnological fields of science and industry. For example, copper nanoparticles biosynthesized from a supernatant obtained after separation of *Bacillus flexus* culture were used to discolor waters containing azodyes used in the textile industry. The described NPs have the potential for discolouration of a wide range of azodyes, such as reactive black–5, Congo red, malachite green, methylene blue, reactive red–2 and direct blue, while the effectiveness of discolouration after 4 h reached 90% or more for some substrates. In addition, it was possible to purify wastewater and reduce the values of a number of pollution indicators (COD, sulfate and phosphate contents) by 50% [100].

To expand the range of biotechnological applications of copper nanoparticles, a biohybrid material preparation with *Clostridium beijerinckii* microorganisms and metal nanoparticles was performed, which was used to improve biobutanol production during the systematic butyrate–butanol fermentation of rice straw. The authors managed to obtain CuS/Cu_2_S, ZnS, and CdS nanoparticles with sizes of 20–30 nm, 15–40 nm, and 15–35 nm, respectively. The use of a biohybrid association based on CuS/Cu2S nanoparticles, in turn, increased the production of biobutanol to 14.6 g/L, which was more than 50% higher than the previously described result of 9.2 g/L [69].

Notably, CuONPs, particularly nanoparticles obtained from microorganisms of the genus *Streptomyces*, have been used in the field of medicine. *Streptomyces capillispiralis* isolated from *Convolvulus arvensis* plants was used to synthesize CuONPs (copper oxide nanoparticles). The obtained nanoparticles demonstrated equally high efficiency in inhibiting the activity of a number of human pathogens: *B. subtilis*, *S. aureus*, *B. diminuta*, *E. coli*, *P. aeruginosa*, *C. albicans* and *A. brasiliensis*. In addition, these nanoparticles inhibited the growth of phytopathogenic fungi, such as *Alternaria alternata* (58.3%), *Fusarium oxysporum* (48%), *Aspergillus niger* (75%) and *Pythium ultimum* (60%), to varying degrees. In addition, nanoparticles have been shown to harm cancer cells and have a larval effect, affecting the mortality of the larvae of houseflies and mosquitoes treated with a solution of *nanoparticles* [73]. Moreover, *Streptomyces* sp. MHM38 microorganisms, in addition to their antibacterial properties, were able to demonstrate antioxidant effects in the absence of biological analogs in relation to liver cells of the studied groups of experimental mice, which also reduced the number of pathologies resulting from oxidative stress [53].

### 4.6. Ni Nanoparticles

Most base metal nanoparticles are widely used in many fields of medicine, including for imparting antibacterial properties to various materials [101,102]. Nickel nanoparticles have many applications, similar to copper nanoparticles, but they are most often used in organic synthesis.

**Figure 16:**
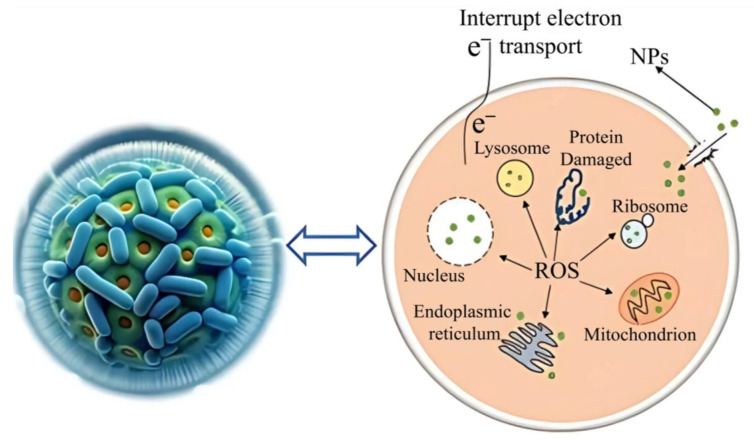
Mechanism of the influence of nickel nanoparticles on fungal cultures [54] ©2024 The authors.

Given the antibacterial activity of nickel nanoparticles, the use of the halobacterium *Halomonas elongata* is worth considering. By using them for biosynthesis, it was possible to produce nanoparticles that effectively reduce the growth of the pathogenic fungus *Candida albicans*, guaranteeing a degree of inhibition of 72.5%, which in turn suggests the possibility of using NiNPs as a coating for dental instruments and an additive to personal oral hygiene products. Moreover, a mechanism for the effect of nanoparticles on pathogenic fungi has been proposed to involve a variety of factors, including the release of reactive oxygen species as a result of peroxidation of the cell wall, destruction of organelles and adsorption of particles on the cell wall, which leads to a deterioration in biofilm formation by these pathogenic fungi (Figure 16) [54].

**Figure 17:**
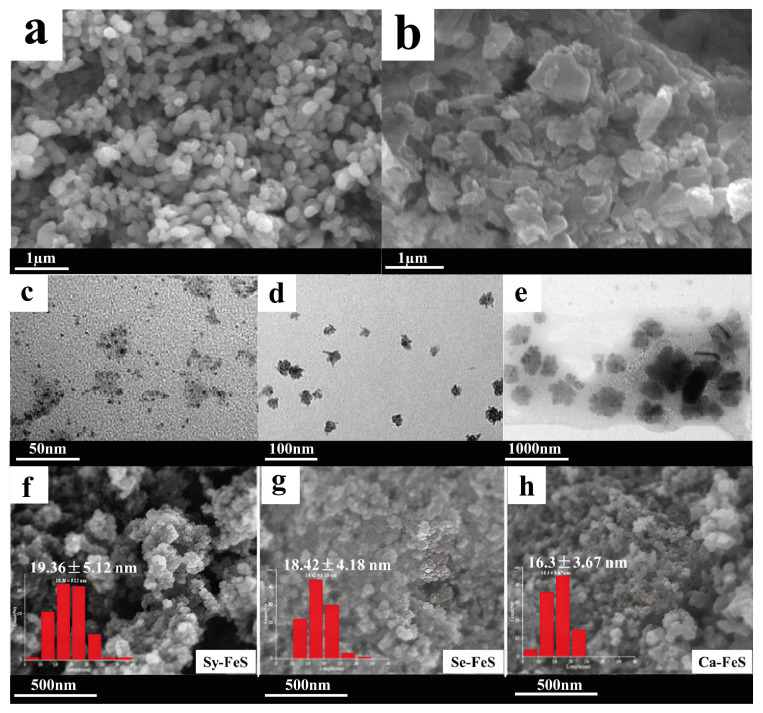
Shapes and sizes of the NPs of various metals: (**a**) SEM image of the NiNPs synthesized with *B. sphaericus* [70] © 2023 SAAB.; (**b**) SEM image of the NiNPs obtained after treatment of industrial wastewater by electroplating via *Microbacterium* sp. MRS-1 [74] © 2014 Elsevier Ltd., Amsterdam, The Netherlands; (**c**) TEM image of the biogenic NiNPs obtained via *P. aeruginosa* SM1; (**d**) TEM image of the biogenic FeNPs obtained via *P. aeruginosa* SM1; (**e**) TEM image of the biogenic CoNPs obtained via *P. aeruginosa* SM1 [75] Springer Science + Business Media B.V. 2012; (**f**) SEM image of the FeSNPs obtained via synchronous biosynthesis (Sy-FeS); (**g**) SEM image of the FeSNPs obtained via sequential biosynthesis (Se-FeS); (**h**) SEM image of the FeSNPs obtained via cathodic biosynthesis (Ca-FeS) [71] © 2023 Elsevier B.V.

Extracellular metabolites of *Bacillus sphaericus* microorganisms, which act as reducing agents and stabilizers, are used in the synthesis of nickel nanoparticles. The resulting particles were rather large (more than 100 nm), while cluster formation by these particles was not mentioned (Figure 17a). The nanoparticles synthesized in this way were studied for their possibility of having a larval effect on the larvae of *Anopheles subpictus* and *Culex quinquefasciatus* mosquitoes, as well as against cattle ticks (*Rhipicephalus annulatus*), which are capable of causing many diseases. Compared with the nickel acetate and cell-free extracts, the obtained nanoparticles were highly effective against both *A. subpictus* and *C. quinquefasciatus*, with an LC_50_ (*A. subpictus*) = 4.29 mg/L and an LC_50_ (*C. quinquefasciatus*) = 4.94 mg/L, respectively [70,103].

Moreover, the bacteria *Microbacterium* sp. MRS-1 was used for the bio-recovery of nickel nanoparticles from electroplating waste, and the microorganisms were cultured on nutrient media containing drains from nickel-galvanic baths. After the microorganisms were cultivated in the presence of nickel salts for five days, the NPs were separated from the cell mass and dried. The nanoparticles obtained by this method had the shape of flakes (Figure 17b), their size varied from 100–500 nm, and the nickel removal coefficient was 90% [74].

The antibacterial properties of nickel nanoparticles obtained from bacteria isolated from Arctic waters and associated with the infusoria *Euplotes focardii* were broader in nature than those of nanoparticles obtained from *H. elongata* and were able to affect both fungi and bacteria. The nanoparticles were most effective against bacteria, as evidenced by the MIC range of 3.12–25 µg/mL, which was 6.25–25 µg/mL for fungi. NPs obtained in this way have been proposed for use in coatings and antiseptic preparations to prevent nosocomial infectious diseases in patients [55].

*Pseudomonas aeruginosa SM1* microorganisms produce nanoparticles of various metals. Most of the obtained nanoparticles were isolated from a cell-free extract. Compared with the iron and lithium nanoparticles, the nickel nanoparticles (Figure 17c), which had a better distribution among the examined ones, had a smaller size, which, despite their good distribution (FeNPs) (Figure 17d), had a larger size. Moreover, lithium nanoparticles (Figure 17e) were localized intracellularly, which, taking into account the particle size (more than 500 nm), is questionable. The formed nanoparticles were localized on the surface, and their size, depending on the metal used, ranged from 2.1 nm to 950 nm [75].

### 4.7. Fe Nanoparticles

Iron nanoparticles are actively used in biotechnology for environmentally friendly synthesis via microorganisms and their enzymes, which ensures particle size and stability control [104]. In medicine, they are used as contrast agents for MRI [105] and for the targeted delivery of antitumor drugs [106] and antibacterial agents [107].

Using biofilms grown with anaerobic activated sludge, FeS nanoparticles were synthesized, as this compound is an effective means of eliminating toxic Cr^6+^. The nanoparticles obtained in this way had a size of no more than 20 nm, despite the formation of many clusters (Figure 17f–h), which may be related to the stabilizing properties of the formed biofilm. Owing to the use of iron sulfide (FeS) nanoparticles in the biocathode of microbial fuel cells, it was possible to increase the current density by 2 times compared with existing studies (up to 42.08 mV/m^2^) and improve the efficiency of eliminating pollutants containing Cr^6+^ to 99.18% [71].

Various forms of iron oxide nanoparticles are widely used in the field of medicine. Thus, Fe_2_O_3_ nanoparticles, which are present in the form of the mineral maghemite, were obtained from a cell-free extract of *Bacillus circulans* bacteria and are subsequently expected to be used as catalysts for the bleaching of dyes and antibacterial agents. The size of the obtained particles was 13.84 nm, whereas owing to calcination at 300 °C, the particles were converted into hematite, the size of which was 23.18 nm. Moreover, studies of antioxidant activity have shown that maghemite particles are more efficient at binding to DPPH (35.44% versus 26.5% for hematite) [56].

Fe_2_O_3_ nanoparticles obtained from *Bacillus megaterium* are proposed for use in hyperthermic therapy. The resulting particles had hexagonal packaging, which ensures the stability of the structure and magnetic properties of the material, which also opens up opportunities for targeted drug delivery [57]. Moreover, *Klebsiella oxytoca* bacteria were able to produce ferrihydrite (5Fe_2_O_3_ · 9H_2_O) nanoparticles. The unstable composition of these nanoparticles, according to assumptions, can affect their content in the active center of the ferritin protein without changing the volume of the inner cavity of the protein itself [76].

### 4.8. Zn Nanoparticles

Zinc nanoparticles are used in many fields of medicine, including as antitumor and antioxidant drugs [108,109]. To optimize the process of obtaining ZnO nanoparticles, *Lactobacillus plantarum* TA4 microorganisms were used, and the effects of 3 factors on the synthesis process were studied: pH, cell-free extract volume, and zinc source concentration. The results of 20 experiments revealed the following optimal parameters for obtaining nanoparticles: 352.4 mM zinc source, 25% by volume of cell-free extract, and pH = 9, which made it possible to achieve an average nanoparticle size of 29.7 nm. Compared with ZnO powder, the obtained nanoparticles showed high overall antioxidant activity and free radical scavenging activity at the same concentrations, with values of 50.1% (10% for ZnO powder) and 99.3% (80% for ZnO powder), respectively [58].

Zinc nanoparticles obtained from *Bacillus subtilis* NH1-8 bacteria were used to inhibit biofilm formation by *Salmonella typhimurium* bacteria. The obtained particles at a concentration of 250 µg/mL inhibited biofouling 86% and reached an inhibition zone of 20 mm at a concentration of 6 mg/mL [59]. Zinc oxide nanoparticles formed intracellularly by another strain of *Bacillus subtilis* bacteria showed relatively high antibacterial activity against *S. typhimurium*, as well as *S. aureus* and *E. coli*. In addition, these nanoparticles were capable of degrading methylene blue: after 30 min, 80% of the dye was removed. [60].

Moreover, the bacteria *Marinobacter* sp. 2C8 and *Vibrio* sp. were isolated from the waters of the Caspian Sea. VLAS contributed to the production of zinc oxide nanoparticles from their cell-free extracts. The particles obtained in this way had different properties and sizes: 10.23 nm and 20.26 nm for 2C8 and VLA, respectively. ZnO-2C8 NPs had greater efficacy against various pathogenic strains and a high degree of inhibition of biofilm formation (96%) at a concentration of 250 µg/mL [61].

## 5. Nanoparticles Synthesized via Plants

Plants and their extracts are widely used as reducing agents in the synthesis of nanoparticles of various metals. In this case, both extracts of whole plants and extracts of specific parts, such as leaves, roots or stems, can be used for synthesis. The main application of such nanoparticles is in the field of medicine or the creation of various antiseptic drugs (Table 2).

The main distinction between nanoparticles synthesized via plant extracts and those synthesized via bacterial methods lies in the diversity of nanoparticle shapes, which results from the greater variety of reducing and stabilizing agents in these extracts. A key advantage of these nanoparticles is their simpler synthesis relative to bacterial methods. Silver nanoparticles (AgNPs) are most commonly produced via this approach because of their excellent antibacterial properties, which are often enhanced through synergistic effects from diverse phytochemicals and organic compounds present in plant extracts. Copper nanoparticles with various shapes (spherical, polygonal, and cubic) derived from plant leaves have been applied in photocatalytic dye degradation and targeted antibiotic delivery. Copper oxides, which are primarily spherical in morphology, have been used for dye adsorption and as fungicides to reduce biofilm formation by the fungus *Candida albicans*. Modern research in the field of nanobiotechnology has demonstrated that plant extracts serve not only as a source of biologically active compounds but also as a powerful tool for the “green” synthesis of metal nanoparticles, allowing the use of various plant species to produce nanoparticles of various metals with desired properties.

**Figure 18:**
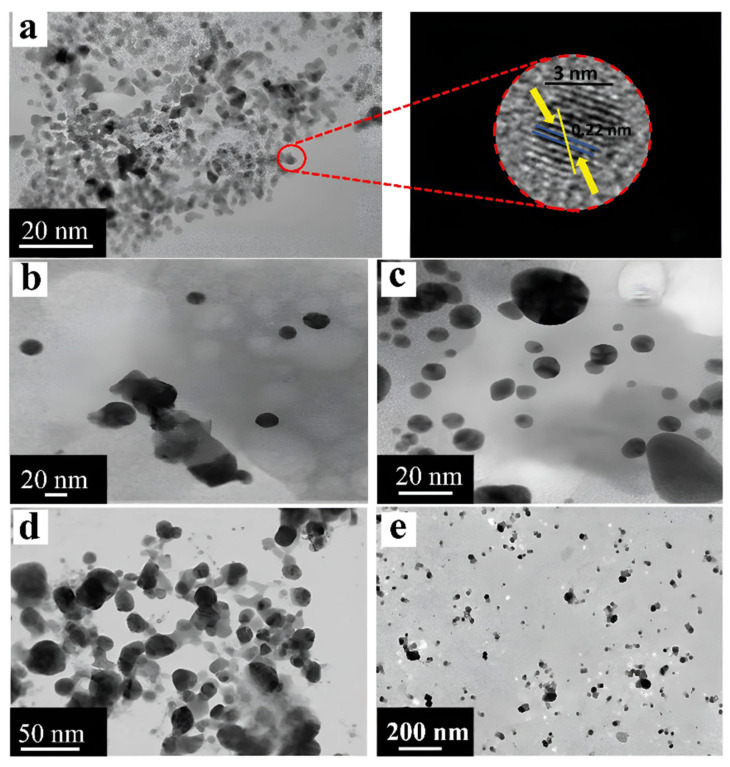
TEM images of the shape and size of precious metal nanoparticles: (**a**) AgNPs obtained from *Eucalyptus citriodora* extract [136] © 2010 Elsevier B.V.; (**b**) AgNPs obtained from *Lysiloma acapulcensis* extract [111] © Authors 2020; (**c**) PtNPs obtained from *Nigella sativa* L. seed extract [112] © 2019 Elsevier B.V.; (**d**) AuNPs obtained from *Jasminum auriculatum* extract [113] © 2020 Elsevier B.V.; (**e**) AuNPs obtained from *Eclipta alba* extract [114] © 2020 Elsevier B.V.

Thus, extracts of eucalyptus (*Eucalyptus citriodora*) and ficus (*Ficus bengalensis*) leaves made it possible to obtain well-dispersed silver nanoparticles (AgNPs) with an average size of 20 nm and a predominantly spherical shape (Figure 18a). Scanning electron microscopy (SEM) confirmed the uniform distribution of AgNPs on the cotton fibers. The antibacterial activity of such materials was tested on *Escherichia coli*: after 7 days, the release of silver ions was 39% for eucalyptus extract and 49% for ficus, which opens up prospects for the creation of functional textile materials with long-lasting antimicrobial action [136].

In addition to *Ficus bengalensis* plants, *Ficus carica* and rosemary *Salvia rosmarinus* plants can be used to produce silver nanoparticles. The aqueous extract of the leaves of these plants made it possible to obtain AgNPs with antibacterial activity. Notably, the authors managed to obtain a colloidal solution of silver nanoparticles by simply mixing the extract and a solution containing a source of silver ions, followed by heating at 60 °C. The AgNPs obtained in this way showed good antibacterial activity against model strains of *E. coli*, *B. cereus*, and *S. aureus* [110].

A mixed extract of the stems and roots of *Lysiloma acapulcensis* plants was also used for the synthesis of AgNPs. The particle size ranged from 1.2 to 62 nm, with a predominance of particles of approximately 5 nm (Figure 18b), which may indicate the formation of agglomerates. Biogenic AgNPs demonstrated high antimicrobial activity against clinical pathogens (*E. coli*, *P. aeruginosa*, *S. aureus*, and *C. albicans*), with a minimum suppressive concentration (MSC) of 0.06 µg/mL for bacteria. Cytotoxicity tests revealed the absence of PI+ lymphocytes, which indicates an early stage of apoptosis that is potentially reversible for cells. This finding indicates a low risk of such AgNPs for the immune system [111].

In addition to their obvious antiseptic and antibacterial applications, precious metal nanoparticles have a wide range of applications. For example, the alcohol extract of *Nigella sativa* L. seeds allows the synthesis of PtNPs with a size of approximately 3 nm (Figure 18c), which exhibit both antibacterial activity against Gram-positive and Gram-negative bacteria and high anticancer efficacy: the inhibition of breast cancer cells (MDA-MB-231) and cervical cancer (HeLa) reached 93% and 96%, respectively [112].

Spherical gold nanoparticles (Figure 18d), which form clusters included in the stabilizing substances contained in the extract of *Jasminum auriculatum* leaves, have been widely applied in many fields. Testing of the antibacterial activity of these nanoparticles has shown that their effectiveness against pathogenic strains is comparable to that of the standard chloramphenicol preparation. With respect to breast cancer cell lines, the same AuNPs inhibited cancer cell activity by 50% at a nanoparticle concentration of 104 mg/mL. In addition to standard tests for antimicrobial and anticancer activity, data from AuNPs have shown their effectiveness in reducing carcinogenic *p*-nitrophenol to *p*-aminophenol in the presence of NaBH_4_, characterized by complete recovery after 45 min [113].

Biogenic gold nanoparticles obtained from *Eclipta alba* extract had an antidiabetic effect. After an alcohol extract from the dried plants was obtained and added to a solution of gold chloride, AuNPs with a size of approximately 26 nm (Figure 18e) were obtained. When the beta cells of the pancreas were exposed to the AuNP solution, a decrease in reactive oxygen species was recorded. Owing to a decrease in the level of Bcl-2 protein and an increase in the level of Bax protein, an increase in the survival of beta cells of the pancreas involved in glucose homeostasis with the help of insulin in the human body has been reported [114].

Through the use of *Simarouba glauca* leaf extract, AuNPs were obtained, which are formed due to the presence of free amino and carbonyl groups capable of reducing Au^3+^ to Au^0^. Using the obtained nanoparticles as antimicrobial drugs, with increasing concentrations of gold salt in the production of nanoparticles, the effectiveness of the AuNPs against model pathogenic strains, such as *Staphylococcus aureus*, *Streptococcus mutans*, *Bacillus subtilis*, *Escherichia coli*, *Proteus vulgaris*, and *Klebsiella pneumonia*, increased, whereas the obtained nanoparticles were more effective against bacteria than the plant extract and the gold salt solution from which the nanoparticles were obtained [115].

Of particular interest are plant extracts rich in biologically active compounds that are able to reduce metal ions to nanoparticles, stabilizing their structure. Citrus fruits (*Citrus* sp.) stand out among such plants, as their peel, juice and other parts contain flavonoids, terpenoids and organic acids, which act as natural reducing agents and stabilizers during the synthesis of nanoparticles [137].

**Figure 19:**
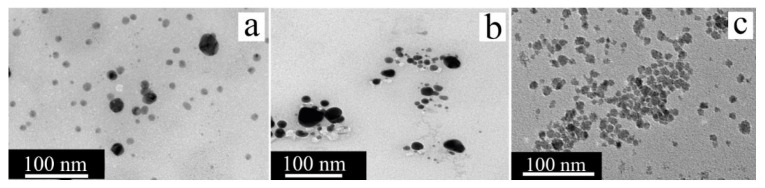
TEM image of the shape and size of the AgNPs and CuNPs; (**a**) AgNPs obtained via *Citrus limetta* peel extract [138] © 2020 Elsevier Ltd., Amsterdam, The Netherlands; (**b**) AgNPs obtained via *Citrus limon* peel extract [116] © 2020 Authors; (**c**) CuNPs obtained via *Citrus sinensis* juice [39] © 2020 Elsevier B.V.

The biogenic AgNPs obtained via *Citrus limetta* peel extract had an average size of 18 nm and did not lose their antibacterial activity for 120 days. In addition to their antibacterial and antifungal properties, these AgNPs have shown the ability to prevent the formation of biofilms and disrupt the permeability of cell membranes. The synthesized well-dispersed AgNPs (Figure 19a) demonstrated high inhibitory ability against several biofilm-forming pathogens (*M. luteus*, *S. aureus*, *E. coli*, *S. mutans*, *S. epidermidis*, *C. glarbrata*, *C. tropicalis*, *C. albicans*, and *C. parapsilosis*) at low concentrations (approximately 6 micrograms/mL). Silver nanoparticles completely suppressed the formation of biofilms of *M. luteus*, *S. aureus* and *E. coli* at concentrations of 10.7 µg/mL, 8.9 µg/mL and 5.9 µg/mL, respectively, which is 4 times lower than that of previously described nanoparticles [138,139].

Silver nanoparticles synthesized from lemon peel extract (*Citrus limon*), with the smallest size of less than 10 nm and an average size of 55 nm, formed clusters larger than 70 nm (Figure 19b). The synthesis process included incubation of the extract with a 1 mM solution of AgNO_3_ at pH 7.0 and 60 °C for 24 h. The bioactive components of the peel, such as limonene, hesperidin and ascorbic acid, play key roles in the reduction of silver ions, which not only reduce Ag^+^ to Ag^0^ but also stabilize the nanoparticles, preventing their aggregation. AgNPs have shown high efficacy against Gram-negative bacteria, including *A. baumannii*, *E. coli*, *P. aeruginosa* and *S. typhimurium*, as well as Gram-positive bacteria, such as *S. aureus* and *P. vulgaris*. The minimum inhibitory concentration (MIC) for *S. aureus* was 8 µg/mL, which is two times lower than that for the antibiotic tetracycline (16 µ/mL). The mechanism of action includes damage to cell membranes due to interactions with thiol groups of proteins and the generation of reactive oxygen species (ROS), leading to oxidative stress. In tests on MCF-7 (breast cancer) and HCT-116 (colon cancer) cell lines, AgNPs reduced cell survival by up to 27% at a concentration of 50 µg/mL. Transcription inhibition is associated with the suppression of RNA polymerase activity, which was confirmed by analysis of the expression of the *MYC* and *BCL2* genes [116].

The copper nanoparticles obtained from *C. sinensis* juice had a spherical shape and a size of 15–40 nm (Figure 19c). The synthesis was carried out at room temperature by adding a 5 mM solution of CuSO_4_ to the filtered juice. Citric and malic acids in the juice were the main reducing agents, forming stable CuNPs due to the chelation of copper ions. CuNPs inhibited the growth of *S. aureus* and *E. coli* at an MIC of 25 µg/mL. The generation of ROS, such as superoxide radicals and hydrogen peroxide, causes the oxidation of membrane lipids, protein denaturation, and breaks in the DNA double helix, which was confirmed by lipid peroxidation tests and COMET analysis. Moreover, the nanoparticles did not have a toxic effect on healthy cells: tests on human fibroblasts (HS-68 line) revealed continued viability even at a concentration of 100 µg/mL. This is due to the selective effect on pathogens and the activation of cellular antioxidant systems such as glutathione peroxidase and SOD in healthy tissues [39].

The spherical CuNPs synthesized from *Citrus medica* Linn. juice had a size of 10–60 nm (average of 33 nm). The bacteria with the highest sensitivity to these nanoparticles were *E. coli* (MIC of 12 µg/mL), *P. acne* (15 µg/mL) and *K. pneumoniae* (18 µg/mL). Among phytopathogenic fungi, CuNPs suppressed the growth of *F. culmorum* by 85%, *F. oxysporum* by 70%, and *F. graminearum* by 65% at a concentration of 50 µg/mL. The mechanism of antifungal action includes the inhibition of the synthesis of ergosterol, a key component of fungal cell membranes. The use of CuNPs in agriculture has demonstrated that treating wheat seeds with a solution of nanoparticles (20 µg/mL) reduces *F. culmorum* damage by 90% and increases germination by 40% [140].

In addition to citrus plants, other fruits and plants that are often found in human life are also used to produce metal nanoparticles. Thus, the fruit of the peach *Nauclea latifolia* served as a reducing agent in the production of biogenic AgNPs. The resulting nanoparticles were studied in creams, which were later used as antibacterial agents for skin infections. The synthesized nanoparticles were tested against a wide range of pathogens: *E. coli*, *Staphylococcus* sp., *S. aureus*, *Klebsiella* sp., *P. aeruginosa*, *C. freundii*, *C. albicans*, *Rhizopus* sp., and *A. niger*. Moreover, *E. coli*, *P. aeruginosa* and *A. niger* were the most sensitive, the area of lysis of which could not be determined because of the high efficiency of AgNPs [141]. Copper nanoparticles were also obtained from green coffee bean extract. The possibility of synthesis was due to the presence of flavonoids and phenolic compounds, which are reducing agents. Nanoparticles 5–8 nm in size were studied for their catalytic activity in the presence of sodium borohydride, and it was shown that within 10–13 min, 90% discoloration of dye solutions occurred due to their decomposition [40].

When nanoparticles are synthesized from plant extracts, the ratio of the volume of the extract to the salt solution is a critical parameter. For example, experiments with *Gomphrena globosa* leaf extract have demonstrated that a decrease in the volume of the extract leads to the formation of smaller AgNPs (15–22 nm) and, as a result, to an increase in their antibacterial activity against pathogens [117].

A number of nanoparticles obtained from *Melia azedarach* leaf extract and *Salvia spinosa* plants grown in vitro were used to prevent the growth of the fungus *Verticillium dahliae* and as antibacterial drugs to eliminate human pathogens, respectively. In the example of infected eggplant seedlings, the high efficacy of AgNPs was demonstrated: in vitro, the study revealed 50% inhibition of mycelial growth compared with the control experiment, and in vivo studies revealed 87% and 97% improvements in the grafted and untreated plants, respectively, compared with the control plants. Moreover, AgNPs obtained from *Salvia spinosa* showed high antibacterial activity against the model pathogens *E. coli*, *B. subtilis*, and *B. vallismortis* due to impaired permeability of the bacterial cell membrane and disruption of key cell life processes, which led to their death [118,119].

In addition to their antibacterial properties, silver nanoparticles obtained from plant extracts are widely used to treat cancerous tumors. Thus, photodynamic therapy for breast cancer was investigated via the use of biogenic silver nanoparticles obtained from *Cynara scolymus* leaf extract. The biogenic AgNPs had an antitumor effect with a semimaximal inhibitory concentration (IC_50_) of 10 mg/mL due to an increase in the production of reactive oxygen species and a decrease in the levels of antioxidant proteins inside diseased cells [120].

In addition to being used to produce silver nanoparticles, extracts of citrus plants are actively used to produce particles of copper and its oxide, which find wide further application. For example, copper nanoparticles obtained from *Jatropha curcas* leaf extract have been shown to discolor organic dyes. Transmission electron microscopy revealed that the particles had an almost spherical shape and an average size of 10 ± 1 nm. Owing to the presence of natural protective and regenerating substances in the plant extract, the nanoparticles remained stable for six months without signs of agglomeration. Using the example of methylene blue, naphthol orange, rhodamine B, and methylene orange, the photocatalytic decomposition ability of dyes under the influence of sunlight and a solution of nanoparticles was considered. The nanoparticles were the most reactive toward methylene blue, and at the same time, they were able to bind to DNA molecules, which would make it possible to detect the binding of drugs to the same DNA molecules via UV spectroscopy [122].

**Figure 20:**
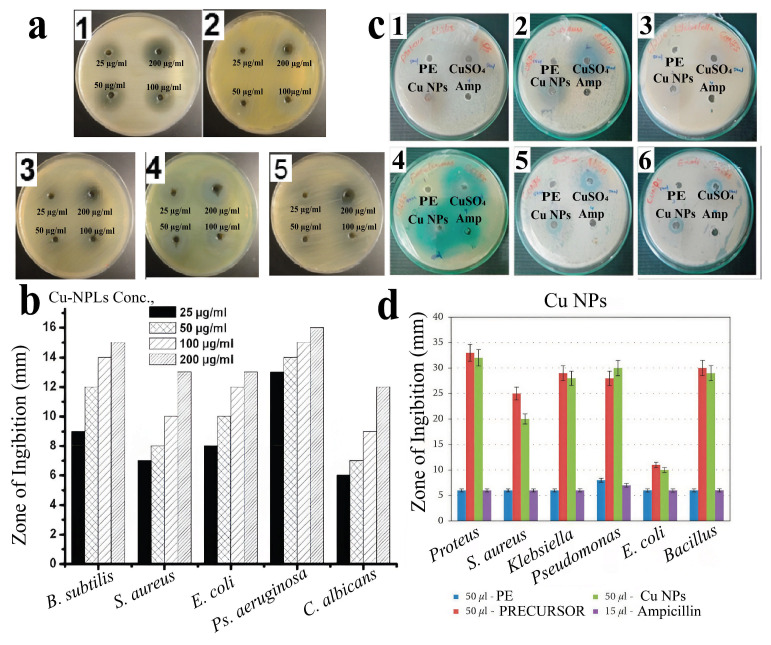
Antibacterial and antifungal activities of the CuNPs: (**a**) antibacterial activity of the CuNP solutions obtained from *Tilia* sp. aqueous extract against (**1**) *B. subtilis*, (**2**) *S. aureus*, (**3**) *E. coli* and (**4**) *P. aeruginosa*; (**5**) antifungal activity of the CuNP solution against *Candida albicans* [121] © 2018 Published by Elsevier Ltd., Amsterdam, The Netherlands; (**b**) lysis zones of the CuNPs obtained from aqueous extracts of *Tilia* sp. leaves against *B. subtilis*, *S. aureus*, *E. coli*, *P. aeruginosa* and *C. albicans*. [121] © 2018 published by Elsevier Ltd., Amsterdam, The Netherlands; (**c**) antibacterial activity of CuNPs obtained via *Andrographis paniculata* leaf extract against (**1**) *Proteus* sp., (**2**) *S. aureus*, (**3**) *Klebsiella* sp., (**4**) *Pseudomonas* sp., (**5**) *E. coli* and (**6**) *Bacillus* sp. [142] © 2021 S. Rajeshkumar et al.; (**d**) lysis zone of CuNPs obtained via *Andrographis paniculata* leaf extract [142] © 2021 S. Rajeshkumar et al.

In addition to their use in photocatalysis, CuNPs can exhibit antibacterial activity, as shown by the example of nanoparticles obtained from extracts of linden leaves (*Tilia* sp.) and andrographis (*Andrographis paniculata*). The sizes of the obtained CuNPs were 4.7–17.4 nm and 42–90 nm for *Tilia* sp. and andrographis, respectively. Notably, the nanoparticles obtained from *Tilia* had a cubic shape, and those obtained from andrography had the shapes of rods, cubes and spheres. At the same time, nanoparticles from both precursors were prone to agglomerate formation but demonstrated high antimicrobial activity (Figure 20) against a wide range of pathogenic microorganisms, showing the greatest effectiveness at a concentration of 200 micrograms/mL (Figure 20a,b) and having efficacy comparable to that of antibiotics and copper sulfate (Figure 20c,d), as well as copper nanoparticles obtained from *Osmium sanctum* leaf extract, which were also used for the controlled release of the antibiotic cobex [38,121,142].

In addition to their use in medicine, CuNPs are also used in ecological applications. For example, copper oxide nanoparticles (CuONPs) synthesized from the extract of pomegranate leaves of *Punica granatum* effectively adsorbed the safranin-O dye. Owing to the negative charge of the CuONP surface, 90% dye removal was achieved at a concentration of 1 g/L. Owing to agglomeration, the rectangular shape of the particles (20–30 nm) did not reduce their efficiency, which makes them promising for wastewater treatment [123].

Interestingly, not only leaves but also flowers can be used to stabilize CuNPs. Thus, the extract of *Bougainvillea flowers*, which are rich in phenols and proteins, made it possible to obtain spherical CuONPs with sizes ranging from 5–20 nm. These particles showed antifungal activity against *Aspergillus niger*, comparable to that of fluconazole. Moreover, the *Duranta erecta* fruit extract made it possible to obtain spherical nanoparticles (~70 nm) that catalyze the reduction of azo dyes. After 4–5 min, they decompose methylene orange (96%) and Congo red (90%), maintaining activity for 4 cycles, albeit with a decrease in the reaction rate [124,143].

The antimicrobial properties of CuNPs have been confirmed by other studies. For example, nanoparticles from the ginger root of *Zingiber officinale*, despite their tendency to agglomerate (size up to 60 nm), inhibited the growth of *S. aureus* and *E. coli*, with an inhibition zone of up to 13 mm. Similar results were obtained for CuNPs from *Dodonaea viscosa* leaves and *Aloe vera* flowers: particles ~40 nm in size suppressed the growth of *E. coli*, *K. pneumonia*, *P. fluorescens*, *S. aureus*, and *B. subtilis* [144,145,146].

Even traditionally edible plants such as *Laurus nobilis* (laurel.) are used in the production of copper nanoparticles. The CuONPs obtained from leaf extracts (90–250 nm) showed antibacterial activity against *E. coli* and *S. aureus*, and 1,8-cineol, linalool, and α–terpinyl acetate were identified as the compounds contributing to this activity. Finally, CuONPs can fight not only bacteria but also fungal biofilms. Particles obtained from the core of *Caesalpinia sappan* suppressed the formation of *Candida albicans* biofilms. Moreover, the particle size (on average, 255 nm) depended on the pH: an increase in the alkalinity of the medium reduced the diameter of the particles [126,127].

The ability of plants to act as reducing agents and stabilizers has also been demonstrated through the synthesis of iron nanoparticles (FeNPs) via *Picrorhiza kurroa* root extract. Particles ~26 nm in size, despite their tendency to agglomerate owing to their magnetic properties, remained stable due to the extract components. The antioxidant activity (51.22%) and antibacterial efficacy of these compounds against *E. coli* and *B. cereus* suggest their potential for the treatment of complex diseases [147].

While FeNPs have potential in fighting bacteria and oxidative stress, zinc oxide nanoparticles (ZnONPs) have opened new horizons in oncology. When synthesized from the endemic *Mentha mozaffarianii* (*Lamiaceae*), ZnONPs with a size of ~29 nm caused the complete death of cervical and breast cancer cells at a concentration of 100 µg/mL. However, the key challenge in the use of ZnONPs is their cytotoxicity. The solution was composed of *Melia azedarach* seed extract: the ZnONPs (47.5 nm) obtained in this way selectively affected hepatoma cells while preserving healthy tissues. Moreover, combining Ag/ZnO particles (~33.5 nm) from the bulbs of *Urginea epigea* expands the possibilities of personalized therapy by combining the properties of silver and zinc [128,148,149].

In addition to zinc, nickel oxide nanoparticles (NiONPs) demonstrate versatility. For example, particles 16–18 nm in size obtained via an extract from *Pedalium murex* not only suppressed pathogens but also contributed to the decomposition of Congo red and rhodamine B dyes by 83–85%, combining medical and environmental objectives. The morphology of the NiONPs directly affects their activity. NPs from *Ocimum tenuiflorum* basil leaves (12–36 nm), having cylindrical, spherical and polygonal shapes, showed increased effectiveness against Gram-negative bacteria and *Candida* fungi [129,130].

*Calendula officinalis* leaf extract was used to enhance antioxidant properties in the production of nickel oxide nanoparticles (NiONPs). Moreover, the average size of the obtained particles was 60.39 nm. The oxide nanoparticles had a spherical shape and, like most nanoparticles produced by “green” chemistry, were prone to agglomeration. These nanoparticles showed impressive results in binding free radicals of 2,2-diphenyl-1-picrylhydrazyl, similar to the results of butyloxytoluene as a standard antioxidant agent. Nickel nanoparticles, depending on the dose, have antitumor effects on esophageal carcinoma without any cytotoxicity to the normal cell line [131].

*Gongronema latifolium* leaves, along with calendula, have been successfully used for the synthesis of spherical nickel oxide (NiO) nanoparticles. These nanoparticles tended toward weak agglomeration. An increase in the concentration of nanoparticles led to a decrease in agglomeration but an increase in their size. The study of antibacterial activity revealed greater activity against the Gram-positive strain of *S. aureus* than against the Gram-negative strain of *E. coli*. Moreover, even toxic plants such as *Euphorbia heterophylla* are becoming sources of useful nanomaterials. Diamond-shaped NiONPs (12–15 nm) obtained from leaves effectively suppressed *E. coli*, confirming that even “dangerous” plant species can be used to produce environmentally friendly and valuable materials [132,134].

The synthesis conditions are another key factor in the production of nickel oxide nanoparticles. Nickel oxide nanoparticles were obtained from the extract of *Lantana camara* flowers. These nanoparticles were obtained by mixing nickel nitrate hexahydrate (Ni(NO_3_)_2_•6H_2_O) with lanthanum flower extract at 60–70 °C for 2 h, after which the solutions were calcined at various temperatures (300, 500, 700 °C) to determine the effects of high temperatures on the shape and formation of clusters of nanoparticles. As the temperature increased, the shape of the nanoparticles became spherical, and their size increased from 14.3 nm at 300 °C to 20 nm and 26 nm at 500 and 700 °C, respectively. Additionally, with increasing annealing temperature, the ability of the nanoparticles to form cluster structures increased. These nanoparticles showed good antibacterial activity against *E. coli*, *S. aureus* and *M. luteus* [133].

In addition to the nanoparticles of metallic nickel and its oxide, the nanoparticles of nickel ferrite NiFe_2_O_4_ were also obtained via *Murayya koenigii* leaf extract. The size of the nanoparticles obtained in this way varied from 2 to 6 nm, and their shape resembled a cubic shape. The ability of the isolated nanoparticles to photocatalytically decompose dyes was tested. The optimal result of 98.5% was achieved in 70 min of wastewater treatment using these nanoparticles. These nanoparticles have good antibacterial properties against various bacterial pathogens, such as *A. faecalis*, *P. aeruginosa*, *S. aureus*, and *E. coli* [135].

## 6. Nanoparticles Synthesized Using Algae and Fungi

In addition to the most common applications of microbial cells for the synthesis of metal nanoparticles, various algae, fungi, or yeasts act as substrates or objects contributing to the synthesis of these same NPs (Table 3).

The use of yeasts, fungi, and algae significantly expands the range of biological reduction agents. The synthesized nanoparticles predominantly exhibit antibacterial activity against human pathogens such as *S. aureus*, *E. coli*, and *P. aeruginosa*. Owing to their small size, nanoparticles derived from fungal extracts particularly stand out. There is a clear relationship between nanoparticle shape and biological source: microalgae (e.g., *Caulerpa racemosa*, *Padina* sp.) produce spherical nanoparticles; fungi (e.g., *Trichoderma asperellum*) form crystalline nanoparticles; yeasts (e.g., *Pichia kudriavzevii*, *Saccharomyces uvarum*) synthesize cubic and rod-shaped nanoparticles. This diversity arises from the unique stabilizing biopolymers of each organism. Certain yeasts, such as *Rhodotorula mucilaginosa* and *Streptomyces* sp. HC1, synthesize specific oxides that inhibit pathogenic biofilm formation, whereas *Saccharomyces uvarum* has synergistic effects with terpenes, increasing oxidative stress in pathogenic microorganisms.

The extract of the green microalga *Botryococcus braunii* was used to synthesize platinum (PtNP) and palladium (PdNP) nanoparticles with various morphologies: cubic, spherical, and truncated triangular. PtNPs had an average size of 86.96 nm, whereas PdNPs had an average size of only 4.89 nm, indicating that the metal influences the nucleation and growth process. Antibacterial disk diffusion tests revealed that PdNPs and PtNPs suppressed pathogens (for example, *E. coli* and *S. aureus*) with an effectiveness comparable to that of ampicillin but 2 times inferior to that of chloramphenicol. Interestingly, the antioxidant activity of the nanoparticles, which was estimated through the binding of DPPH free radicals, reached 82.43% for PdNPs and 78.14% for PtNPs at concentrations ranging from 20–25 µg/mL. Although these values are lower than those of ascorbic acid (94%), they demonstrate the potential of nanoparticles in combating oxidative stress [150].

Silver nanoparticles synthesized with *Botryococcus braunii* go beyond antimicrobial applications. AgNPs have been used as catalysts in the eco-friendly one-pot synthesis of benzimidazoles, which are key structures for anticancer and antifungal drugs. The method allowed the synthesis of 9 derivatives with yields of up to 80%, minimizing the use of toxic reagents. For example, the reaction with ortho-phenylenediamine and benzaldehyde gave 2-phenylbenzimidazole in 75% yield, confirming the effectiveness of this approach for “green” pharmaceuticals. However, synthesis without stabilizers is fraught with risks. *Botryococcus braunii*, which reduces Ag^+^ and Cu^2+^ salts, forms clusters of nanoparticles due to the lack of stabilizing agents. Despite this, the AgNPs obtained from this microorganism showed record toxicity: the MIC for *S. aureus* was 8 µg/mL (versus 32 µg/mL for chloramphenicol), and the inhibition zone for *P. aeruginosa* reached 18 ± 1.2 mm. This is due to the synergy between the nanoparticles and antimicrobial metabolites of algae, such as botryococcene [151,152].

**Figure 21:**
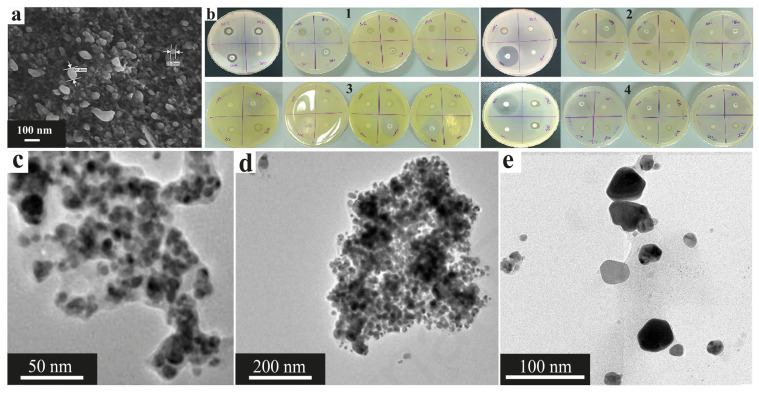
Shape and size of various precious metal nanoparticles: (**a**) SEM image of AgNPs synthesized as a result of the reaction of *Padina* sp. extract with 0.01 M silver nitrate (AgNO_3_) solution [153] © Authors. 2020; (**b**) antibacterial activity against four different concentrations of AgNPs, positive control and negative control: 1—inhibition zone formed by the Gram-positive bacteria *Staphylococcus aureus*; 2—inhibition zone formed by the Gram-positive bacteria *Bacillus subtilis*; 3—inhibition zone formed by the Gram-negative bacteria *Pseudomonas aeruginosa*; 4—inhibition zone formed by the Gram-negative bacteria *Escherichia coli* [153] © The Authors. 2020; (**c**) TEM image of Ag_2_ONPs synthesized from *Trichoderma harzianum* IB-363 [158] © Authors 2020; (**d**) TEM image of AuNPs obtained from the *Ganoderma lucidum* mushroom extract [160]; (**e**) TEM image of AuNPs obtained from *P. kudriavzevii* HA-NY2 [161] © Springer-Verlag GmbH Germany, part of Springer Nature 2021.

The antibacterial properties of AgNPs have been studied in detail via the example of *Padina* sp. seaweed. Particles with a size of 33.75 nm (Figure 21a) stabilized with decanoic acids from the extract showed different activities against Gram-positive and Gram-negative bacteria: the inhibition zone for *S. aureus* (Gram-positive) was 15.17 ± 0.58 mm, and that for *P. aeruginosa* (Gram-negative) was 13.33 ± 0.76 mm (Figure 21b). The reduced effectiveness against Gram-negative bacteria is due to their complex cell wall; however, AgNPs have surpassed traditional antiseptics as antibiofouling agents [153].

The AuNPs obtained from *Caulerpa racemosa* were 20–50 nm in size and exhibited dual antibacterial and antitumor activities. Thus, they inhibited 50% of human adenocarcinoma cells at a concentration of 20.84 µg/mL (MTT test, 24 h) because the mechanism of action is associated with the induction of apoptosis through the activation of caspase-3/9. In parallel, AuNPs suppressed *S. agalactiae* (lysis zone 23.96 mm) and *A. veronii* (20.14 mm), which makes them promising for combination therapy for cancer and infections [154].

In addition to being used in the synthesis of nanoparticles for medical applications, algae can be used to solve environmental problems. *Chlamydomonas reinhardtii* not only removes copper ions from wastewater (bioadsorption up to 90% in 48 h) but also synthesizes spherical CuNPs (5–6 nm). Organic ligands (for example, cell wall polysaccharides) stabilize the particles, preventing their oxidation and agglomeration. These CuNPs can be used in catalysis or as antimicrobial coatings. In addition, the stability of the CuNPs depends on the composition of the extract. The protein fraction of *Macrocystis pyrifera*, which is rich in thiol groups and aromatic compounds, allows the synthesis of CuONPs up to 50 nm in size. Interestingly, the F3 fraction (molecular mass of 30–50 kDa) had the smallest particle size (12 ± 3 nm), which was correlated with a high content of cysteine-containing peptides that chelated copper ions [155,156].

If algae demonstrate potential in the controlled synthesis of metal oxides, then fungi can create ultrafine nanoparticles with unique properties. For example, extracellular synthesis using *Trichoderma viride* made it possible to obtain AgNPs (2–4 nm) in size. The incubation of fungi at 40 °C for 48 h activated enzymes (e.g., NADH-dependent reductases) and low-molecular-weight metabolites (organic acids), which reduced Ag^+^ to Ag^0^. Owing to surface plasmon resonances and quantum effects, the luminescence of AgNPs in the 320–520 nm range makes them promising for creating biosensors or optical nanomaterials [41].

Another type of *Trichoderma* mushroom, *Trichoderma asperellum*, is relevant for the treatment and prevention of cancer. The synthesized CuONPs (110 nm) inhibited cancer cells by increasing the level of reactive oxygen species (ROS) by 35%. This leads to DNA fragmentation and a decrease in the mitochondrial potential, triggering apoptosis. The crystal structure of the particles enhances their reactivity, which is critical for therapeutic applications. The versatility of the *Trichoderma harzianum* fungus was confirmed by the synthesis of nanoparticles (Figure 21c) of Ag, CuO and ZnO (size 58.87–582.40 nm). Ag and CuO nanoparticles suppressed the phytopathogens *Alternaria alternata* and *Pyricularia oryzae*, slowing mycelial growth by 60–80% at a concentration of 100 µg/mL. In contrast, ZnO is ineffective because of differences in the mechanisms of action: Ag and CuO generate ROS, whereas ZnO requires UV activation to manifest toxicity [157,158].

In turn, widespread *Candida albicans* fungi also contribute to the synthesis of gold and silver nanoparticles. AuNPs (4–10 nm) and AgNPs (30 nm) synthesized by this fungus showed different antibacterial activities: AgNPs suppressed *E. coli* more effectively (inhibition zone 18 ± 1.5 mm) than did AuNPs (12 ± 0.8 mm). This is due to the ability of silver to disrupt the thiol groups of enzymes and denature proteins, whereas gold acts through electrostatic interactions with membranes [159].

The morphology of the nanoparticles may depend on the synthesis method. The cell-free extract of *Aspergillus sojae* fungi was used for the biosynthesis of cobalt and zinc oxide nanoparticles, which were then used as antibacterial agents because of their effectiveness against food-borne bacterial pathogens. *Aspergillus sojae* produces Co_3_O_4_ (floccular) and ZnO (oval) nanoparticles, which form clusters up to 500 nm in size. Despite agglomeration, Co_3_O_4_ had a rather low MIC value of 1.25 mg/mL against *S. aureus*, which is twice as effective as MIC ZnO. This is due to the ability of cobalt to disrupt the transport of electrons in the respiratory chain of bacteria [162]. Moreover, the TiO_2_-doped ZnO nanoparticles obtained from *Aspergillus niger* were rods (500–1000 nm in length). Owing to interactions with cytosolic proteins, longer rods are produced via intracellular synthesis, whereas extracellular particles are shorter (300–600 nm). Agglomeration is explained by the removal of biomass, which disrupts the stabilization of fungal polysaccharides [42].

An example of synthesis optimization is the study of silver nanoparticles obtained from *Rhizopus stolonifera*. AgNPs with a size of 2–15 nm (average 9.46 ± 2.64 nm) were formed within 48 h because of the peptides and organic acids in the extract [163]. The result of such optimization processes was a combined approach to nanoparticle synthesis using *Ganoderma lucidum*, which made it possible to create AgNPs with dual functions. At a concentration of 100 µg/mL, the particles cleaved bacterial DNA within 60 min and inhibited breast cancer cells (IC_50_ = 45 µg/mL). The synergy between mushroom terpenoids (e.g., ganaderic acid) and AgNPs (Figure 21d) enhances oxidative stress in pathogenic microorganisms [160].

The gold nanoparticles obtained via the extract of the edible morel *Morchella esculenta* were 16.51 nm in size and had pronounced bioactive properties. The antioxidant activity of gold nanoparticles, which are highly effective against pathogenic microorganisms and fungi, as well as lung and liver cancer cell lines, was studied. With an increase in the concentration of AuNPs, an increase in antioxidant activity occurred, which was shown by the example of the binding of iron ions and the absorption of 2,2-diphenyl-1-picrylhydrazyl (DPPH) and linoleic acid by β-carotene. At a concentration of 10 mg/mL, the activity was 82, 85, and 77% for binding iron ions, taking up DPPH, and β-carotene-linoleic acid, respectively [164].

Like fungi such as *Morchella esculenta*, which have potential in the synthesis of multifunctional nanoparticles, yeast also opens new possibilities for the controlled synthesis of metals and their oxides. For example, a cell-free extract of the yeast *Stereum hirsutum* made it possible to obtain Cu/CuO nanoparticles with a size of 4 nm. Under optimal conditions (neutral pH, CuCl_2_, weekly cultivation), NADH-dependent reductases are activated, resulting in the reduction of copper ions. Despite the duration of the process and the mixed phase (Cu/CuO), such particles are promising for catalytic applications, although additional optimization is required to obtain them in a stable form [167].

The antimicrobial potential of copper has increased due to the possible use of CuO nanoparticles (14 nm) obtained from *Streptomyces* sp. MHM38 extract. When proteins (for example, metallothioneins) are extracted, the particles are stabilized, preventing their agglomeration. CuO showed unequal activity against bacteria and fungi: the MIC for *C. albicans* was 8 µg/mL, whereas for *E. coli*, it was 32 µg/mL. This is due to the ability of copper to disrupt the thiol-dependent enzymes of fungi, which are critical for their metabolism [53].

The yeast *Rhodotorula mucilaginosa*, which was isolated from seawater and coastal soils, was selected because it is potentially capable of synthesizing Cu_2_O nanoparticles. These NPs have been used as a means of fighting cancer cells. The synthesized nanoparticles exhibited intracellular localization, and their average size was 51.6 nm for optimally selected conditions (2.5 mM CuSO_4_, pH = 7). The cytotoxic effects of the NPs were tested on 6 types of cancer cells, and the semimaximal inhibitory concentrations ranged from 10.7 µg/mL to 108 µg/mL [165].

Titanium dioxide is actively used in medicine as an antibacterial stainless coating, particularly in surgery. On the basis of these properties, via the use of *Streptomyces* sp. HC1 yeast, TiO_2_NP nanoparticles ranging in size from 30 to 70 nm were obtained, which were further investigated as antibacterial drugs. The antipathogenic properties were evaluated via model pathogenic microorganisms, yeast and fungi. Studies have shown that the formed nanoparticles are more effective against bacterial pathogens than against yeast and fungi but also exhibit an antifilm effect against *P. aeruginosa* microorganisms. The maximum zone of inhibition was observed for *S. aureus* and *E. coli*. Moreover, the maximum activity against biofilms was observed when 500 µL of nanoparticles was used [166].

Using two strains of fermentable yeast, *Pichia kudriavzevii* and *Saccharomyces uvarum*, AgNP nanoparticles were obtained extracellularly. The resulting nanoparticles were round and cubic in shape and had sizes of 12.4 nm and 20.7 nm for *S. uvarum* and *P. kudriavzevii*, respectively (Figure 21e). The silver nanoparticles synthesized from *S. uvarum* were smaller and had greater anti-inflammatory and antitumor activities than those synthesized from *P. kudriavzevii*. Compared with antibiotics, AgNPs have been shown to be more effective against inflammatory processes in carrageenan-induced edema in rats. In addition, they are also effective against prostate and intestinal cancer cell lines and have demonstrated antimicrobial activity against several important pathogens, including *S. aureus*, *E. coli*, *C. tropicalis*, and *F. oxysporium* [161].

## 7. Comparison of the Efficiency of Catalytic Applications of Metal Nanoparticles

The biological source, metal used, and localization of biogenic nanoparticles—and thus their accessibility to reaction substrates—significantly alter their catalytic properties. Diverse metabolites and structural features from bacteria and yeasts [21,24,25], along with peptide biopolymers from plants, algae, and fungi [26,31,32] present in biological materials, provide the primary advantage of excellent catalytic performance to these nanoparticles.

Platinum nanoparticles demonstrate high efficiency in reducing toxic Cr(VI) compounds. The biomaterial used alters nanoparticle size and accessibility, with smaller particles (16.1 nm from *A. aromatica* and 28.9 nm from *A. cryptum*) being more effective [20]. Copper nanoparticles are effective in multiple catalytic reactions, including azide–alkyne cycloaddition (20–50 nm from *S. oneidensis*) [23] and the degradation of various azo dyes [100]. Both CuNPs synthesized via bacteria and those produced with plant extracts exhibit high efficiency in these processes [40]. CuO nanoparticles [67] and Au nanoparticles [82] effectively reduce the dangerous carcinogen *p*-nitrophenol to harmless *p*-aminophenol in the presence of NaBH_4_, albeit with varying degrees of efficiency. Palladium nanoparticles remain the most versatile and catalyze the aforementioned reduction of *p*-nitrophenol [85] and Cr(VI) [64]. Biogenic palladium nanoparticles have been widely applied in cross-coupling reactions, particularly the Suzuki–Miyaura and Mizoroki–Heck reactions. PdNPs produced by various bacteria exhibit varying catalytic activities, likely due to differences in the accessibility of catalytically active centers, which can reduce the range of suitable substrates from 15 to 3 [66]. Biogenic nanoparticles of various metals are widely applicable in numerous catalytic reactions, comparable to their classically synthesized counterparts. Owing to their biological origin, these nanoparticles often outperform chemical analogs under identical conditions [62,63].

## 8. Similarities and Differences in Antimicrobial Mechanisms of Biogenic Metal Nanoparticles

The antimicrobial activity of metal nanoparticles is a multifactorial process involving multiple mechanisms of damage to microbial cells. Various nanoparticles can have antimicrobial effects both in accordance with universal mechanisms and through various metal-specific mechanisms of antimicrobial action. Universal mechanisms include the generation of reactive oxygen species, which is similar for most metal nanoparticles [39,54]; increased permeability of cell membranes due to a violation of their integrity [119,138]; and disrupting the replication mechanism of cell DNA [160].

Considering metal-specific mechanisms, it is worth starting with the most common metal used in this way—silver. The antimicrobial effect of silver nanoparticles is largely due to the gradual release of Ag^+^ ions, which interact with the thiol groups (-SH) of enzymes and proteins, causing their denaturation [168]. Thus, AgNPs from psychrophilic and mesophilic bacteria (*P. antarctica*, *A. kerguelensis*, *A. gangotriensis*, and *B. indicus*) demonstrated pronounced antimicrobial activity, as did nanoparticles obtained using *Candida albicans* fungi. This approach was more effective than the use of Au nanoparticles obtained from the same source, which can be explained by the greater effectiveness of the thiol-binding mechanism of action of the AgNPs than the electrostatic action of the AuNPs [47,159].

The mechanism of action of copper nanoparticles is the Cu^2+^/Cu^+^ conversion cycle, which allows the generation of hydroxyl ions via the Fenton mechanism [169]. Thus, cultures of bacteria of the genus *Stenotrophomonas* have made it possible to obtain nanoparticles effective against human pathogens and phytopathogens, which are also able to inhibit the growth of biofilms of pathogenic microorganisms through destruction of the polysaccharide matrix forming them [50,96]. In addition to these metals, other metal nanoparticles also have antimicrobial properties: zinc oxides are also able to inhibit biofilm growth in a similar way to CuNPs and cobalt and nickel nanoparticles because of their ability to disrupt the respiratory processes of cells in the electron transport chain [43,55,61].

The composition of the cell wall of the inhibited microorganisms also affects the effectiveness of the nanoparticles. For example, silver nanoparticles were obtained with the help of the algae extract *Padina* sp. Different efficacies were shown relative to those of Gram-negative (*P. aeruginosa*) and Gram-positive (*S. aureus*) bacteria, which demonstrated a decrease in the zone of inhibition of Gram-negative microorganisms, which can be explained by the more complex structure of the cell wall [153]. In turn, NiONPs from *Gongronema latifolium* showed great activity against the Gram-positive *S. aureus* strain than against the Gram-negative *E. coli* strain [132].

The mechanisms of antifungal activity are somewhat different from those of antibacterial activity: CuNPs from *Citrus medica* Linn suppressed the growth of phytopathogenic fungi by inhibiting the synthesis of ergosterol, a key component of fungal cell membranes, and AgNPs from *Melia azedarach* effectively prevented the growth of the fungus *Verticillium dahlia*, and both types of metal nanoparticles improved the growth characteristics of various plants (CuNPs—wheat; AgNPs—eggplant seedlings) [118,140].

Importantly, antimicrobial effects are often the sum of several factors, which can be expressed as various synergistic effects. In combination with antibiotics, AgNPs obtained using a cell-free extract of the cyanobacterium *Anabaena variabilis* demonstrated a synergistic effect, manifested as a significant decrease in the concentration of nanoparticles and antibiotics necessary to suppress the growth of pathogens [49]. AgNPs obtained from the extract of the algae *Botryococcus braunii*, owing to the metabolites contained in the extract, inhibited *P. aeruginosa* several times more effectively than *S. aureus* did [152].

## 9. Challenges and Perspectives

Despite the numerous advantages of the biosynthesis of metal nanoparticles via microorganisms and plant extracts, this field of research faces a number of significant technological and scientific challenges that need to be addressed, including the successful transition from laboratory research to industrial production. Particle agglomeration remains the primary challenge: biosynthesized nanoparticles tend to form agglomerates despite their many advantages. This is critically important for their use as heterogeneous catalysts for organic synthesis reactions. This problem is especially relevant in synthesis without sufficient natural stabilizers; for example, *Botryococcus braunii* algae, which reduce Ag^+^ and Cu^2+^ salts, form clusters of nanoparticles due to a lack of effective binding agents [152], and *Aspergillus sojae* fungi produce Co_3_O_4_ and ZnO nanoparticles that form clusters up to hundreds of nanometers in size [43]. The control of the size and stability of nanoparticles is critically important for their catalytic activity, as shown by the example of palladium nanoparticles synthesized by *Cupriavidus metallidurans*: particle aggregation (>500 nm) was observed in the presence of formate, whereas the addition of glutaraldehyde made it possible to obtain a stable colloid (10–20 nm) [64]. It is important to overcome poor reproducibility of results through standardization, which is especially difficult for plant extracts due to seasonal changes, and for bacteria, strict observance of cultivation conditions and culture age is important. This problem can be solved by introducing strict standardization of the technological process, which, in the future, may pave the way for the transition from laboratory testing to industrial processes. The scaling of such processes is the main obstacle to the introduction of such methods in production. This transition is associated with the slow kinetics of biogenic processes, the difficulty of maintaining homogeneous conditions in large bioreactors, and the need for fine control over the supply of substrate and oxygen [170,171]. Intracellular synthesis creates additional difficulties in isolating nanoparticles from the cellular matrix, which makes purification processes multistage and resource intensive. A number of strains, such as *Rhodococcus erythropolis* ATCC4277, require long cultivation periods (7–14 days) to accumulate sufficient concentrations of reducing metabolites, which significantly slows the overall production cycle. These factors explain why biogenic nanoparticles are rarely used in industrial processes and often find their application in narrow fields [98]. Equally important is the question of the safety of using these nanoparticles, especially in clinical trials during the study of their antitumor and antioxidant properties. The toxicity of nanoparticles depends on the size, shape, chemical composition, and functionalization of the surface, including the charge, surface area, and ability to generate reactive oxygen species. Studies have shown that biogenic AgNPs can cause cytotoxic and genotoxic effects due to increased oxidative stress, DNA damage, and the induction of apoptosis, and the severity of these effects significantly depends on the size distribution and concentration of the particles [172,173]. For a number of systems, there is no toxicity to particles on the order of 10–20 nm at low doses, and there is a noticeable increase in toxicity with increasing size or a change in surface chemistry [64]. These data emphasize the need for standardized safety assessment protocols for biogenic nanomaterials, comparable to the approaches used for chemically synthesized nanoparticles: cytotoxic assays (MTT assays) and standard physicochemical studies (TEM/SEM, BET analysis, XPS, etc.) [174].

The introduction of machine learning and artificial intelligence at various stages of the synthesis process is proposed as the main way to improve and simplify the synthesis of biogenic metal nanoparticles. For example, owing to the intervention of AI, it will be possible to reduce the number of experimental experiments aimed at optimizing synthesis conditions, as well as using these tools to predict the possible structure and properties of particles on the basis of a large number of existing experimental data described in the scientific literature [175,176].

In terms of the possible prospects for further application of such nanoparticles, this is the development of approaches for bioremediation and waste recycling. Thus, the *Pseudomonas stutzeri* strain, which is used for the utilization of copper ore tailings with the simultaneous formation of CuNPs [72], has the potential to combine bioremediation and synthesis of nanoparticles. The use of electronic waste, such as printed circuit boards, as a source of copper for the production of CuO-NPs with the participation of *Alcaligenes aquatilis* opens the way for the creation of closed metal circulation cycles and reduces the environmental burden [67]. In addition to waste processing, biogenic nanoparticles are actively considered for water purification from heavy metals, dyes, and organic toxicants, including Cr(VI) reduction, degradation of azo dyes, and detoxification of *p*-nitrophenol [64,65,122]. The most important aspect of studying the processes of biorecovery of metals and the production of nanoparticles is the establishment of synthesis mechanisms in various groups of microorganisms, as well as the factors influencing the processes occurring inside cells. The use of molecular biology methods that can control the production of certain metabolites or increase cell survival under the influence of the produced nanoparticles is possible.

## 10. Conclusions

The biosynthesis of metallic nanoparticles via microorganisms, plants, fungi, algae, and yeasts represents a promising and environmentally friendly approach for producing nanoparticles of gold, silver, platinum, palladium, copper, nickel, iron, and other metals. These nanoparticles have controllable sizes (ranging from 1 to 100 nm), predominantly spherical or crystalline morphologies, and high stability due to the use of biological stabilizers. They exhibit remarkable catalytic activity, often surpassing chemically synthesized analogs in cross-coupling reactions (Suzuki–Miyaura, Mizoroki–Heck); the reduction of hazardous materials, such as *p*-nitrophenol, Cr(VI), and dyes; and pronounced antimicrobial properties against pathogens such as *E. coli*, *S. aureus*, and *C. albicans*, including biofilm inhibition and synergism with antibiotics.

The key advantages of green synthesis include low toxicity, the possibility of using waste materials (electronic or mining) for remediation, and the multifunctionality of nanoparticles in medicine (anticancer and antioxidant effects), agriculture, and biotechnology (yield enhancement, biobutanol production). However, challenges remain, including particle agglomeration, low reproducibility owing to biomaterial variability, difficulties in scaling up, and the need to standardize parameters such as pH, temperature, and precursor concentration. We believe that future prospects of green nanobiotechnology are associated with the optimization of biohybrid catalysts, their combination with other nanomaterials, and the transition to industrial-scale production, which would enable the realization of the full potential of biogenic nanoparticles in sustainable chemistry, medicine, and environmental applications.

## Figures and Tables

**Table 1:** Characteristics of metal nanoparticles obtained with the help of microorganisms and their cell-free extracts.

Nanoparticles	Microorganisms	Nanoparticle Size, nm	Nanoparticle Shape	Localization of Nanoparticles in the Cell	Application of Nanoparticles	Literature
**Nanoparticles used in the field of biomedicine**						
Ag	*Pseudomonas antarctica* *Pseudomonas proteolytica* *Pseudomonas meridiana* *Arthrobacter kerguelensis* *Arthrobacter gangotriensis* *Bacillus indicus* *Bacillus cecembensis*	3.4–33.6 2.8–23.1 2.2–21.5 4.2–26.1 3.6–22.8 2.5–13.3 2.8–18.2	spherical	extracellular	an antibacterial drug to counteract pathogenic microorganisms	[47]
Ag	*Bacillus subtilis*	3–20	spherical	obtained using a cell-free extract	prevention of infectious diseases, inhibition of bacterial biofilm growth	[48]
Ag	*Anabaena variabilis*	11–15	spherical	obtained using a cell-free extract	an antibacterial drug to counteract pathogenic microorganisms	[49]
Cu	*Stenotrophomonas maltophilia*	18.5–31.7	spherical	cell surface	antibacterial drug for combating phytopathogens and human pathogens, decomposition of pesticides	[50]
Cu	*Shewanella loihica*	6–20	spherical	cell surface	inactivation of *Escherichia coli* cells	[51]
Cu	*Bacillus cereus*	11–33	spherical	-	an antibacterial drug to counteract pathogenic microorganisms, anticancer drug	[52]
CuO	*Streptomyces* sp.	1.06–6.54	spherical	obtained using a cell-free extract	reduction in oxidative stress, liver protection, antibacterial drug to counteract pathogenic microorganisms	[53]
Ni	*Halomonas elongata*	22	spherical	obtained using a cell-free extract	coating for dental instruments	[54]
Ni	*Marinomonas* sp. *ef1*, *Rhodococcus* sp. *ef1, Pseudomonas* sp. *ef1*, *Brevundimonas* sp. *ef1*, *Bacillus* sp. *ef1*	20–50 15–50 20–50 20–50 20–50	rod-shaped, spherical	cell surface, intracellular	an antibacterial drug to counteract pathogenic microorganisms	[55]
α-Fe_2_O_3_ γ-Fe_2_O3	*Bacillus circulans*	23.18 13.84	irregular agglomerated clusters	obtained using a cell-free extract	antioxidant drug	[56]
Fe_2_O_3_	*Bacillus megaterium*	20–30	spherical	obtained in the total volume of biomass	a drug for the treatment of hyperthermia	[57]
ZnO	*Lactobacillus plantarum TA4*	29.7	spherical	obtained using a cell-free extract	antioxidant drug	[58]
ZnO	*Bacillus subtilis NH1-8*	39	hemispherical	obtained using a cell-free extract	inhibition of bacterial biofilm growth	[59]
ZnO	*Bacillus subtilis*	451	spiky flakes	obtained using a cell-free extract	an antibacterial drug to counteract pathogenic microorganisms, the catalyst for the degradation of methylene blue	[60]
ZnO	*Marinobacter* sp. *2C8* *Vibrio* sp. *VLA*	10.23 20.26	hexagonal	obtained using a cell-free extract	an antibacterial drug to counteract pathogenic microorganisms, inhibition of bacterial biofilm growth	[61]
**Nanoparticles used in catalysis**						
Pt	*Acidocella aromatica* *Acidiphilium crytpum*	16.1 28.9	spherical	cell surface	catalysis of the Cr(VI) to Cr(III) reduction reaction	[20]
Pd	*Paracoccus yeei*	4.9 3.7 2.8	spherical	cell surface between cells in the sarcina intracellular	catalysis of cross-coupling reactions (Suzuki–Miyaura, Mizoroki–Heck)	[62]
Pd	*Paracoccus yeei*	3.99 (a) 9.1 (b) 10.7 (c)	spherical	cell surface and intracellular (a) living cells (b) pasteurized cells (c) autoclaved cells	catalysis of cross-coupling reactions (Suzuki–Miyaura, Mizoroki–Heck)	[63]
Pd	*Cupriavidus metallidurans*	2–40	spherical	cell membrane	catalysis of the Cr(VI) to Cr(III) reduction reaction and reduction of p-nitrophenol	[64]
Pd	*Enterococcus faecalis*	<10	spherical	cell membrane, intracellular	catalysis of the Cr(VI) to Cr(III) reduction reaction	[65]
Pd	*Cupriavidus necator* *Pseudomonas putida*	~10	spherical	periplasmic membrane	catalysis of cross-coupling reactions (Suzuki–Miyaura, Mizoroki–Heck)	[66]
Cu	*Shewanella oneidensis*	20–50	spherical	intracellular	the catalyst of the azide-alkyne addition reaction	[23]
Cu	*Alcaligenes aquatilis*	23.5	spherical	obtained using a cell-free extract	catalytic reduction of p-nitrophenol to p-aminophenol	[67]
**Nanoparticles used in other fields/without specifying the field of application**						
Cu	*Kocuria flava*	10–30	spherical	obtained using a cell-free extract	increasing crop yields	[68]
CuS/Cu_2_S ZnS CdS	*Clostridium beijerinckii*	20–30 15–40 15–35	spherical	obtained using a cell-free extract	optimization of lignocellulose butanol production	[69]
Ni	*Bacillus sphaericus*	23	spherical	obtained using a cell-free extract	larvicidal drug against mosquito larvae and ticks	[70]
FeS	Anaerobic activated sludge	20	spherical	obtained using a cell-free extract	improving the efficiency of Cr^6+^ elimination in microbial fuel cell biocathodes	[71]
Cu	*Pseudomons stutzeri*	1–5	-	cell surface	-	[72]
Cu	*Bacillus* sp.	0.19	-	intracellular	-	[19]
Cu	*Streptomyces capillispiralis*	3.6–59	spherical	cell surface	-	[73]
Ni	*Microbacterium* sp. *MRS-1*	100–500	flakes	obtained using a cell-free extract	-	[74]
Ag Pd Fe Rh Ni Ru Pt Co Li	*Pseudomonas aeruginosa*	6.3 22.1 205 2.1 2.9 8.3 450 550 950	spherical polygonal flakes polygonal polygonal spherical spherical flakes polygonal	obtained using a cell-free extract, intracellular (Co, Li)	-	[75]
5Fe_2_O_3_ · 9H_2_O	*Klebsiella oxytoca*	3–5	spherical	obtained in the total volume of biomass	-	[76]

**Table 2:** Characteristics of metal nanoparticles obtained via plant extracts.

Nanoparticles	Plant	Part of the Plant	Nanoparticles Size, nm	Nanoparticles Shape	Nanoparticles Application	Literature
Ag	*Ficus carica* *Salvia rosmarinus*	leaves	14–31 20–63	spherical	antibacterial drug to counteract human pathogens	[110]
Ag	*Lysiloma acapulcensis*	whole plant	1.2–62	crystal, spherical quasispherical	antibacterial drug to counteract human pathogens	[111]
Pt	*Nigella sativa* L.	seeds	3.47	spherical	antibacterial drug to counteract human pathogens, anticancer drug	[112]
Au	*Jasminum auriculatum*	leaves	8–37	spherical	antibacterial drug to counteract human pathogens	[113]
Au	*Eclipta alba*	whole plant	26	spherical	antidiabetic drug	[114]
Au	*Simarouba glauca*	leaves	>10	prismatic, spherical	antibacterial drug to counteract human pathogens	[115]
Ag	*Citrus limon*	peel	55.84	spherical	antibacterial drug to counteract human pathogens, anticancer drug	[116]
Ag	*Gomphrena globosa*	leaves	5 mL: 15.64 10 mL: 19.44 15 mL: 22.16	polygonal	antibacterial drug to counteract human pathogens	[117]
Ag	*Melia azedarach*	leaves	18–30	spherical	fungicide	[118]
Ag	*Salvia spinosa*	whole plant	19–125	spherical	antibacterial drug to counteract human pathogens	[119]
Ag	*Cynara scolymus*	leaves	98.47	spherical	photodynamic therapy of breast cancer	[120]
Cu	*Tilia* sp.	leaves	4.7–17.4	spherical, hemispherical	antibacterial drug to counteract human pathogens	[121]
Cu	*Jatropha curcas*	leaves	11–12	spherical	photocatalytic decomposition of dyes	[122]
Cu	*Osmium sanctum*	leaves	3.9–10.9	cubic, spherical	targeted delivery of the antibiotic kobex	[38]
Cu	*Citrus sinensis*	leaves	6.93–20.70	spherical	antibacterial drug to counteract human pathogens	[39]
Cu	Green coffee	beans	5–8	polygonal	photocatalytic decomposition of dyes	[40]
CuO	*Punica granatum*	leaves	20–30	rectangular	removal of the safranin-O dye by adsorption	[123]
CuO	*Bougainvillea*	flowers	8–16	spherical	antifungal drug	[124]
CuO	*Canthium coromandelicum*	leaves	33	spherical	-	[125]
CuO	*Laurus nobilis*	leaves	90–250	spherical	antibacterial drug to counteract human pathogens	[126]
CuO	*Caesalpinia sappan*	core	255	spherical	a drug that prevents the formation of biofilms by *Candida albicans* yeast	[127]
ZnO	*Mentha mozaffarianii*	leaves	29	spherical	anticancer drug	[128]
NiO	*Pedalium murex*	leaves	16–18	polygonal	antibacterial drug to counteract human pathogens	[129]
Ni	*Ocimum tenuiflorum*	leaves	12–36	polygonal	antibacterial drug to counteract human pathogens	[130]
NiO	*Calendula officinalis*	leaves	60.39	spherical	anticancer drug that counteracts esophageal carcinoma	[131]
NiO	*Gongronema latifolium*	leaves	-	spherical	antibacterial drug to counteract human pathogens	[132]
NiO	*Lantana camara*	flowers	14.3–26	spherical	antibacterial drug to counteract human pathogens	[133]
NiO	*Euphorbia heterophylla*	leaves	12–15	diamond-shaped	antibacterial drug to counteract human pathogens	[134]
NiFe_2_O_4_	*Murayya koenigii*	leaves	2–6	cubic	antibacterial drug to counteract human pathogens	[135]

**Table 3:** Characteristics of metal nanoparticles obtained from microalgae, fungi, and yeast.

Nanoparticles	Biomaterial	Nanoparticles Size, nm	Nanoparticles Shape	Nanoparticles Application	Literature
Pd Pt	*Botryococcus braunii*	4.89 89.96	cubic, spherical, truncated-triangular	antibacterial drug to counteract human pathogens, antioxidant drug	[150]
Ag	*Botryococcus brauni*	88.87	cubic	-	[151]
Cu Ag	*Botrycoccus braunii*	40–100 10–70	Ag: cubic, spherical, truncated-triangular Cu: cubic, spherical, elongated	antibacterial drug to counteract human pathogens	[152]
Ag	*Padina* sp.	25–60	spherical	antibacterial drug to counteract human pathogens	[153]
Au	*Caulerpa racemosa*	13.7–85.4	spherical	anticancer drug, antibacterial drug to counteract human pathogens	[154]
Cu	*Chlamydomonas reinhardtii*	5–6	spherical	-	[155]
CuO	*Macrocystis pyrifera*	2–50	spherical	-	[156]
Ag	*Trichoderma viride*	2–4	spherical	-	[41]
CuO	*Trichoderma asperellum*	110	crystal, spherical	anticancer drug	[157]
CuO Ag_2_O ZnO	*Trichoderma harzianum*	58.87–582.40	spherical	antibacterial drug to counteract human pathogens	[158]
Au	*Candida albicans*	4–10	spherical	antibacterial drug to counteract human pathogens	[159]
Ag	*Ganoderma lucidum*	15–22	spherical	antibacterial drug to counteract human pathogens	[160]
Ag	*Pichia kudriavzevii* *Saccharomyces uvarum*	12.4 20.7	spherical, cubic	anticancer drug, antibacterial drug to counteract human pathogens	[161]
CuO	*Aspergillus terreus*	>25	spherical	antibacterial drug to counteract human pathogens	[162]
ZnO	*Aspergillus niger*	500–1000	rod—shaped	antibacterial drug to counteract human pathogens	[42]
Ag	*Rhizopus stolonifer*	6.04	spherical	-	[163]
Au	*Morchella esculenta*	16.51	spherical	antibacterial drug to counteract human pathogens	[164]
CuO	*Streptomyces* sp. MHM38	14	spherical	antibacterial drug to counteract human pathogens	[53]
Cu_2_O	*Rhodotorula mucilaginosa*	51.6	spherical	anticancer drug	[165]
TiO_2_	*Streptomyces* sp. HC1	30–70	spherical	antibacterial drug to counteract human pathogens	[166]
Co_3_O_4_ ZnO	*Aspergillus sojae*	500	cereals, oval-shaped	antibacterial drug to counteract human pathogens	[43]

## Data Availability

No new data were created or analyzed in this study.
